# Hereditary Cancer Syndromes: A Comprehensive Review with a Visual Tool

**DOI:** 10.3390/genes14051025

**Published:** 2023-04-30

**Authors:** Mattia Garutti, Lorenzo Foffano, Roberta Mazzeo, Anna Michelotti, Lucia Da Ros, Alessandra Viel, Gianmaria Miolo, Alberto Zambelli, Fabio Puglisi

**Affiliations:** 1 CRO Aviano, National Cancer Institute, IRCCS, 33081 Aviano, Italy; 2 Department of Medicine, University of Udine, 33100 Udine, Italy; 3 Unit of Oncogenetics and Genomics CRO Aviano, National Cancer Institute, IRCCS, 33081 Aviano, Italy; 4 Medical Oncology and Hematology Unit, IRCCS—Humanitas Research Hospital, Rozzano, 20089 Milan, Italy; 5 Department of Biomedical Sciences, Humanitas University, Pieve Emanuele, 20072 Milan, Italy

**Keywords:** hereditary cancer syndromes, cancer predisposition syndromes, cancer genetics, BRCA, lynch syndrome, Li–Fraumeni, melanoma, breast cancer, colon cancer

## Abstract

Hereditary cancer syndromes account for nearly 10% of cancers even though they are often underdiagnosed. Finding a pathogenic gene variant could have dramatic implications in terms of pharmacologic treatments, tailored preventive programs, and familiar cascade testing. However, diagnosing a hereditary cancer syndrome could be challenging because of a lack of validated testing criteria or because of their suboptimal performance. In addition, many clinicians are not sufficiently well trained to identify and select patients that could benefit from a genetic test. Herein, we searched the available literature to comprehensively review and categorize hereditary cancer syndromes affecting adults with the aim of helping clinicians in their daily clinical practice through a visual tool.

## 1. Introduction

Hereditary cancer syndromes (HCSs) account for nearly 10% of cancers even though they are often underdiagnosed [1,2]. Arising from mutations that confer an elevated susceptibility to cancer development, HCSs typically exhibit an autosomal dominant inheritance pattern, with a 50% risk of transmission to offspring. Although each syndrome displays highly specific clinical manifestations, there are common indicators that can aid in their identification, such as the early onset of cancer, the occurrence of multiple tumors in the same individual, a positive family history, and an atypical sex distribution (for example, breast cancer in a man).

Genetic variants, the molecular basis of HCSs, describe DNA sequence variations within an individual’s or population’s genome that can influence gene expression or function to varying degrees. The American College of Medical Genetics and Genomics (ACMG) has established a framework to classify genetic variants into five categories based on their pathogenicity: Pathogenic (P), Likely pathogenic (LP), Uncertain significance (VUS), Likely benign (LB), and Benign (B) [3]. This classification scheme allows clinicians to interpret the significance of genetic variants and tailor patient management accordingly. In particular, while variants classified as pathogenic or likely pathogenic are considered clinically actionable and warrant disease-specific surveillance and/or management, VUS can present a significant challenge as their effect on gene expression or function is unclear, leaving it uncertain whether they contribute to the development of a genetic syndrome and requiring careful monitoring and genetic counseling to determine whether the VUS is clinically actionable or not. 

Finding a gene variant predisposing to cancer in a patient through a genetic test could have at least three profound implications for his/her clinical scenario: 

First, some gene variants are predictive of cancer treatment efficacy; for example, *BRCA1/2* for PARP inhibitors or the mismatch repair system for immune checkpoint inhibitors. In such instances, a genetic test could dramatically increase the therapeutic armamentarium for that patient [4,5].

Second, finding a HCS in a patient allows a tailored preventive program to be offered that might increase his/her lifespan with a favorable cost-to-benefit ratio, such as organ specific surveillance and prophylactic surgeries [6,7].

Third, the same gene variant can be searched for in every family member, expanding the benefit burden of therapeutic and preventive strategies discussed above [8,9].

However, the diagnosis of a HCS could be challenging because of a lack of validated testing criteria or because of their suboptimal performance [10,11]. In addition, many clinicians are not sufficiently well trained to identify and select patients that could benefit from a genetic test. 

Herein, we searched the available literature to comprehensively review and categorize HCSs affecting adults (Table 1). We discuss in greater detail some HCSs linked to specific organs. In addition, we generated a visual tool to help clinicians to find a putative HCS quickly and easily in each patient through the personal/familiar history evaluation (Table 2 and Table S1). 

## 2. HCS of Renal Cancer

Renal cancers can be associated with genetic syndromes, the most relevant of which are von Hippen–Lindau Syndrome (VHLS), hereditary papillary renal carcinoma syndrome (HPRC), hereditary leiomyomatosis and renal cancer cell syndrome (HLRCC), and Birt-Hogg-Dubé syndrome (BHD) (Table 1, Table 2 and Table S1).

VHLS is caused by inactivation of the VHL protein (pVHL), whose best characterized function is the targeting of hypoxia inducible factor (HIF) for proteosomal degradation. Although rarely the first presentation of disease, up to 80% of patients with VHL develop renal cell carcinoma (specifically, clear cell carcinoma) [12]. Classic associated clinical signs are flank pain, hematuria, and/or a palpable mass in the flank, but an abdominal CT scan remains the best diagnostic approach [13]. Contextually, other types of cancer may occur in patients with VHLS. Up to 30% of cases are associated with pheochromocytoma, up to 12% with pancreatic neuroendocrine tumors, and up to 4% with endolymphatic sac tumors. Associated benign/preneoplastic lesions include hemangioblastomas of the central nervous system (often the first manifestation of disease), retinal capillary hemangioblastomas, multiple and bilateral renal cysts, paragangliomas, endolymphatic sac tumors of the middle ear, pancreatic serous cystadenomas, and epididymal and broad ligament cystadenomas. VHL syndrome is classified into several types, but among all of them, type 2b, which is more common in European countries, is characterized by the highest risk of developing clear cell renal cell carcinoma.

HLRCC is a rare disorder caused by a germline mutation in the *fumarate hydratase* (*FH*) gene that encodes an enzyme required in the Krebs cycle to convert fumarate to malate. HLRCC predisposes to various renal tumors, such as type 2 papillary renal cell cancer, collecting duct renal cell carcinoma, clear cell renal cell carcinoma, and Wilm’s tumor, which is present in 19% of all cases and often characterized by being unilateral and solitary but with exceptional aggressiveness. Other malignancies that characterize HLRCC syndrome include cutaneous and uterine leiomyosarcomas, Leydig cell tumors, gastrointestinal stromal tumors (GIST), and breast cancer. The outstanding feature of HLRCC, however, is the occurrence of multiple cutaneous leiomyomas, originating from the hair erector pili muscles and typically localized in limbs, trunk, and face [14]. Other benign tumors can be found not infrequently, such as uterine leiomyomas, which are typically numerous (from 1 to 20), large (up to 10 cm in diameter) and associated with menorrhagia, irregular menses and pain; ovarian cystadenomas; pheochromocytoma; and paraganglioma [14]. 

BHD, caused by germline mutations in the tumor suppressor gene *FLCN* (encoding the protein folliculin, involved in cell growth), is associated with renal tumors of various histologic types in up to 19% of cases: hybrid oncocytic/chromophobe tumor, chromophobe tumor, oncocytoma, clear cell carcinoma, papillary carcinoma, and mixed-type carcinoma. Numerous are the potentially associated clinical manifestations. At the skin level, numerous characteristic benign lesions are present, such as fibrofolliculomas, skin tags (acrochordons), angiofibromas, and oral papules, although the risk of developing cutaneous (including multiple desmoplastic melanomas) and choroidal melanoma is not negligible. More than 80% of patients develop multiple bilateral lung cysts, usually asymptomatic or associated with cough or exertional dyspnea but predisposing to spontaneous pneumothoraxes with a high recurrence rate [15]. Rarer is the occurrence of pulmonary adenocarcinoma. BHD syndrome predisposes to numerous other malignant tumors, such as colon, thyroid/parathyroid, and parotid cancer (see Table 2) and benign tumors in soft tissues, colon, the musculoskeletal system, and kidney (see Table 2). 

Hereditary papillary renal cell carcinoma (HPRC) is another inherited renal cancer syndrome, associated with pathogenic variants of the *MET* gene, that predisposes patients to bilateral and multifocal papillary type 1 renal tumors.

Other syndromes with less frequency predispose to renal tumors, including the Tuberous Sclerosis Complex (TSC) with a 4% lifetime risk (lft), Li–Fraumeni syndrome (LF), Bloom syndrome (BSyn), Fanconi Anemia, BAP1 tumor predisposition syndrome (BAP1) with up to 10% lft, Lynch syndrome (LS) with up to 16% lft, *MUTYH*-associated polyposis (MAP), and hereditary diffuse gastric cancer syndrome (HDGC) with up to 10% lft.

## 3. HCS of Thyroid Cancer

Among the genetic syndromes associated with thyroid tumors, Multiple endocrine neoplasia type 2 (MEN2A and MEN2B) and Cowden syndrome (CS) are the most relevant (Table 1, Table 2 and Table S1).

Multiple endocrine neoplasia type 2 (MEN2) is an inherited cancer syndrome associated with germline variations of the *RET* proto-oncogene, which encodes a tyrosine kinase receptor whose activation promotes cell growth, proliferation, and differentiation. MEN2 is clinically divided into two entities: MEN2A and MEN2B. Medullary thyroid carcinoma (MTC), present in 90% of MEN2A and 100% of MEN2B cases, is often the first manifestation of disease, with a mean age of diagnosis ranging from 19 to 33 years [16]. 

A typical feature of MEN2A is the occurrence, with a prevalence of 17–42%, of both unilateral and bilateral pheochromocytoma. More easily bilateral than sporadic, typical symptoms of pheochromocytoma are hypertension, headache, sweating, and tachycardia [17]. Associated benign/preneoplastic lesions include parathyroid adenoma/hyperplasia, C-cell hyperplasia (CCH), cutaneous lichen amyloidosis with characteristic lesions at the scapular site, and Hirschsprung disease (HSCR), often diagnosed shortly after birth. 

MEN2B is characterized by early and aggressive development of MTC, often associated with metastatic disease at an early age [17]. Its many distinctive benign features include C-cell hyperplasia (CCH), mucosal neuromas (lips, tongue, pharynx, palate), submucosal nodules (lips), and ganglioneuromatosis of the gastrointestinal tract which leads to gastrointestinal symptoms such as diarrhea and constipation, distinctive facies with enlarged lips and marfanoid habitus. Abnormalities of the central nervous system include cerebellar dysplastic gangliocytoma (adult Lhermitte–Duclos disease), intracranial vascular malformations, macrocephaly, intellectual disabilities, and autism. Characteristic mucocutaneous lesions include facial trichilemmomas, acral keratoses on the extensor sides of the extremities, papillomatous lesions, and mucocutaneus neuromas. Gastrointestinal involvement is frequent (up to 80%, according to recent literature), with colorectal and gastrointestinal polyps (often hamartomas), and esophageal glycogen acanthosis. Other benign lesions that can be found include fibrocystic disease of the breast, lipomas, fibromas and macular pigmentation of the glans penis, genitourinary malformation, testicular lipomatosis, and uterine fibroids.

Although less frequently than the above syndromes, Familial adenomatous polyposis (FAP, caused by a germline mutation in the *Adenomatous Polyposis Coli (APC)* gene) can also be associated with the occurrence of thyroid tumors in up to 12% of patients. However, its prominent feature remains the appearance, during the second decade of life, of hundreds to thousands of colorectal adenomas which almost inevitably progress to carcinoma [18]. Benign extraintestinal manifestations that may occur include gastric fundic gland polyps, duodenal adenomas, congenital hypertrophy of the retinal pigment epithelium (CHRPE), juvenile nasopharyngeal angiofibromas, osteomas, dental abnormalities such as supernumerary teeth and odontomas, epidermoid or sebaceous cysts, and benign thyroid nodules. 

CS is discussed below. Other syndromes with less frequency, including Attenuated familial adenomatous polyposis (AFAP), DICER1, and the Carney complex (CNC), predispose to thyroid tumors.

## 4. HCS of Melanoma

There are numerous genetic syndromes that result in an important predisposition to the occurrence of melanoma: Familial atypical multiple mole melanoma (FAMMM), Xeroderma pigmentosum (XP), BAP1 tumor predisposition syndrome (BAP1), Shelterin complex genes hereditary cancer syndrome (Shelterin), MC1R polymorphism (MC1R), and DICER1 (Table 1, Table 2 and Table S1).

Patients with FAMMM (mainly associated with alteration of the *CDKN2A* gene, involved in cell cycle regulation), clinically characterized by the presence of atypical nevi and a total body nevi count > 50, have a very high risk of melanoma, ranging from 28% to 67%. Other potentially associated typical malignancies include pancreatic cancer (adenocarcinoma, up to 17%), astrocytoma, breast cancer, lung cancer, head and neck, and gastroesophageal cancer, the latter three apparently only in smoking patients. 

Another prominent syndrome is Xeroderma pigmentosum (XP), caused by alterations in genes involved in the nucleotide excision repair system and associated with skin cancer (basal cell carcinoma, squamous cell carcinoma, melanoma) in up to 65% of cases. XP is clinically characterized by increased sensitivity to the sun, typically associated with severe sunburn with blistering, persistent erythema, xerosis, and poikiloderma, and by sunlight-induced ocular involvement, such as photophobia, keratitis, and atrophy of the skin of the lids. In about 25% of patients, there is progressive neurodegeneration with sensorineural hearing loss, acquired microcephaly, progressive cognitive impairment, and diminished deep tendon stretch reflexes. Other malignant diseases that characterize Xeroderma pigmentosum include oral cancer, sarcoma, hematological diseases such as myelodysplastic syndrome and acute leukemia, colorectal and non-small cell lung cancer, glioma, and others (see Table 2).

BAP1 tumor predisposition syndrome (BAP1) is a newly recognized cancer syndrome that predisposes to uveal and cutaneous melanoma (36% and 12–45%, respectively). BAP 1 is a deubiquitinating enzyme that acts as a tumor suppressor protein, regulating cell cycle, cellular differentiation and DNA damage repair. Uveal melanoma, which is the most frequently found cancer in these patients, has a mean age of onset of 51 years, lower than the 61 in the general population, and is typically more aggressive, with a high risk of metastasis and reduced overall survival [19]. In contrast, cutaneous melanoma can be found as a single or multiple skin lesion, is associated with greater aggressiveness, and appears earlier than in the general population [19]. BAP syndrome can also predispose to a wide range of other cancers, including, for example, malignant mesothelioma (25%), renal cell carcinoma (10%), and cholangiocarcinoma (1.4%). Benign lesions associated with the BAP1 mutation include multiple cutaneous melanocytic neoplasms called “BAP1-inactivated melanocytic tumors” and meningiomas.

Germline mutations in the Shelterin family of genes (*ACD*, *TERF2IP*, *TERF1*, *TERF2*, *TINF2*, and *POT1*), overall collected in the Shelterin complex genes hereditary cancer syndrome (Shelterin), can cause hereditary melanoma [20] along with chronic lymphocytic leukemia, glioma, angiosarcoma (in particular cardiac angiosarcomas), and colorectal and thyroid cancer. 

In addition, individuals with *MC1R* polymorphism, clinically characterized by red hair/fair skin, have a higher melanoma risk, together with squamous cell and basal carcinoma. Furthermore, the epistatic effect of *MC1R* on *CDKN2A* accounts for the markedly increased risk of melanoma development in individuals carrying both genetic alterations.

*MITF*, a member of the *Myc* gene family, encodes a transcription factor that regulates the differentiation and proliferation of melanocytes. A germline variant of *MITF* (p.E318K) has been shown to be predisposing to melanoma, with a 3–5-fold increase in associated risk compared with the normal population [21,22]. Individuals with variants of *MITF*, clinically characterized by darker hair, fair skin, and non-blue eye color [22], are contextually predisposed to the occurrence of uterine carcinosarcoma, renal cancer, pheochromocytoma, and pancreatic cancer. 

Other syndromes that are less frequently associated with the occurrence of melanoma include the LF syndrome and the TERT hereditary cancer syndrome (TERT), the latter associated with somatic promoter mutations of *TERT*, considered to be one of the earliest secondary mutations following *BRAF* and *NRAS* driver mutations [23].

## 5. HCS of Breast, Ovarian, Pancreatic, and Prostate Cancer

Considering the hereditary breast cancer landscape, there are several genetic syndromes correlated to it (Table 1, Table 2 and Table S1). The most frequent syndromes related to higher risk of developing breast cancer are LF (up to 85%; gene: *TP53*) [24,25]; *BRCA1*- and *BRCA2*-associated hereditary cancer syndrome (lft 69–72%; gene: *BRCA1/2*) [26,27,28,29]; *STK11*-associated hereditary cancer syndrome or Peutz–Jeghers syndrome (PJS) (up to 54%; gene: *STK11*) [30,31,32]; *PALB2*-associated hereditary cancer syndrome (up to 53%; gene: *PALB2*) [28,29,33,34,35,36,37,38]; *ATM*-associated hereditary cancer syndrome or Ataxia telangiectasia (lft 30–52%; gene: *ATM*) [28,38,39,40]; hereditary diffuse gastric cancer syndrome (HDGC) (up to 42%; gene: *CDH1*) [41,42,43]; *BARD1*-associated hereditary cancer syndrome (up to 40%; gene: *BARD1*) [28,38,44]; Multiple endocrine neoplasia type 1 (up to 33%; gene: *MEN1*) [45,46]; *CHEK2*-associated hereditary cancer syndrome (up to 30%; gene: *CHEK2*) [28,38,47,48,49]; Cowden Syndrome (CS) (up to 30%; gene: *PTEN*) [26,50,51]; *RAD51C*-associated hereditary cancer syndrome (up to 21%; gene: *RAD51C*) [28,29,38,52]; *RAD51D*-associated hereditary cancer syndrome (up to 20%; gene: *RAD51D*) [28,38,52,53]; Neurofibromatosis type 1 (up to 18%; gene: *NF1*) [54,55]; Fanconi Anemia (FA, several genes; see Table 1) [56,57,58,59]; Polymerase proofreading-associated polyposis (PPAP; gene: *POLE, POLD1*) [60,61,62,63]; *NTHL1* tumor syndrome (gene: *NTHL1*) [64,65,66]; Bloom syndrome (BSyn; gene: *BLM*) [67]; and *MLH3*-associated polyposis (gene: *MLH3*) [68].

Beyond genetic analysis, differential diagnosis among these syndromes is possible, considering the different characteristic phenotypes of each syndrome. In this context, we could look for particular features corresponding to diagnostic criteria (often divided into major ones and minor ones for each syndrome) but also findings suggestive of or possibly associated with a specific syndrome. 

In this syndromes group, we could find Ataxia telangiectasia, PSJ, FA, Polymerase proofreading-associated polyposis, *NTHL1* tumor syndrome, CS, HDGC, *MLH3*-associated polyposis, NF1, MEN1, LF, and BSyn. 

Ataxia telangiectasia could present into three different forms: heterozygotic/monoallelic, classic ataxia telangiectasia (homozygotic/biallelic), and the non-classic form (attenuated phenotype). In this syndrome, the altered gene is *ATM*, which encodes ATM, a serine/threonine protein kinase involved in DNA damage detection. In the biallelic form, it is characterized by neurological problems such as progressive cerebellar ataxia or progressive dystonia, while in heterozygosis, coronary heart disease is a frequent manifestation [39,40]. 

The presence of multiple hamartomatous polyps, especially gastrointestinally, is peculiar to PSJ. In addition to this, people with PSJ present with freckling or hyperpigmentation on the lips, buccal mucosa, genitalia, fingers, and toes. In this case, the *STK11* gene encodes the Serine/Threonine Kinase 11 (STK11), which is involved in several functions: DNA damage response, apoptosis, cellular metabolism, and polarity. It acts as a tumor suppressor protein, controlling the activity of AMP-activated protein kinase (AMPK) family members [30,31,32].

Considering FA, more than 20 responsible genes have been correlated with it (FANCA, FANCC, FANCD1/BRCA2, FANCD2, FANCE, FANCF, FANCG, FANCI, FANCJ/BRIP1, FANCL, FANCM, FANCN/PALB2, FANCP/SLX4, FANCQ/ERCC4, FANCR/RAD51, FANCS/BRCA1, FANCT/UBE2T, FANCU/XRCC2, FANCV/REV7, FANCW/RFWD3, and FANCY/FAP100). FA-related genes encode proteins which work together in “the FA-pathway”, a DNA damage response pathway. FA can typically be identified by congenital abnormalities such as those included in the VACTERL-H (Vertebral, Anal, Cardiac, Tracheo-esophageal fistula, Esophageal atresia, Renal, upper Limb and Hydrocephalus) association. In addition, Fanconi Anemia is often characterized by endocrine disorders, skin pigmentation alterations, growth deficiency, skeletal and ophthalmic malformations, central nervous system alterations, and hematological abnormality [56,57,58,59]. 

CS is associated with epithelial thyroid cancer (usually follicular, rarely papillary, never medullary) in up to 10% of cases. Other malignancies that characterize CS include genitourinary cancer (especially renal cell carcinoma, up to 10%), endometrial cancer (10%), colorectal cancer (9%), and melanoma (6%). The CS phenotype is peculiar for cerebellar dysplastic gangliocytoma, macrocephaly, mucocutaneous lesions, hamartomatous intestinal polyps, and genitourinary malformation. CS is characterized by alterations in the *PTEN* gene, which encodes the protein PTEN, a phosphatidylinositol-3,4,5-trisphosphate 3-phosphatase. This protein negatively regulates the PI3K/AKT/mTOR pathway [26,50,51]. 

BSyn is caused by a mutation in the gene *BLM*, encoding the DNA helicase RecQ protein that is involved in several pathways for the maintenance of genome stability. BSyn has a typical phenotype: proportionate small stature, facial and skin pigmentation alterations (sun-sensitive facial erythema), immunodeficiency, diabetes mellitus, variable intellectual impairment, hypogonadism, and premature menopause [67]. 

NF1’s typical presentations are cutaneous features (mainly café au lait macules, intertriginous freckling, plexiform/nodular neurofibromas), ocular alterations (i.e., optic pathway gliomas, Lisch nodules), neurologic alterations, musculoskeletal disorders, and vascular and cardiac problems. NF1-responsible alterations occur in the *NF1 (Neurofibromin 1)* gene, which encodes neurofibromin, a protein belonging to a family of guanosine triphosphate hydrolase (GTPase)-activating proteins (GAPs) that negatively regulates the Ras signal transduction pathway [54,55]. 

The MEN1-related gene is *MEN1 (Menin 1)* which encodes menin, a scaffold protein that functions in epigenetic gene regulation. MEN1 is known for three main characteristics (neuroendocrine tumors, parathyroid and pituitary adenomas, pheochromocytomas), which may also include skin alterations as well as thyroid disorders [45,46]. 

Cleft lip/palate is a particular feature of hereditary diffuse gastric cancer syndrome; in breast cancer, this syndrome typically is associated with a specific histotype, namely, lobular breast cancer. This syndrome is caused by alterations in *CDH1* (*Cadherin 1*), a gene encoding CDH1, a calcium-dependent cell adhesion protein. CDH1 regulates the proliferation and mobility and of epithelial cells and cell–cell adhesions [41,43,54,69].

Multiple colonic adenomas are characteristic of PPAP (0–100 cumulative polyps) [60,61,62,63], *NTHL1* tumor syndrome (1–100 cumulative polyps) [64,65,66], *MLH3*-associated polyposis (10–100 cumulative polyps) [68], and LF (colorectal polyps) [24,25]. In PPAP, duodenal adenomas are another typical characteristic, as they are for the MDPL syndrome (mandibular hypoplasia, deafness, progeroid features, and lipodystrophy) (*POLD1*) [60,61,62,63]. Considering this group of syndromes, related genes code for different proteins. For PPAP, *POLD1* encodes the catalytic subunit of DNA polymerase delta, while *POLE* encodes the catalytic subunit of DNA polymerase epsilon; both these proteins are involved in genome replication [60,61,62,63]. In the *NTHL1* tumor syndrome, the altered gene is *NTHL1 (Nth Like DNA Glycosylase 1*), encoding a DNA N-glycosylase (endonuclease III family) involved in base excision repair (BER) [64,65,66]. *MLH3 (MutL Homolog 3)* is the altered gene in *MLH3*-associated polyposis, and the encoded protein MLH3 plays a role in maintaining genomic integrity during DNA replication and after meiotic recombination [68]. LF is related to a mutation in *TP53*, also known as the “guardian of the genome”. The encoded protein is a tumor suppressor protein involved in inducing cell cycle arrest, DNA repair, senescence, apoptosis, and changes in metabolism [24,25].

The *NTHL1* tumor syndrome also presents with duodenal adenomas, meningiomas, skin alterations, ovarian and hepatic cysts, and breast papillomas [64,65,66].

On the other hand, genetic syndromes with alterations in *BRCA1/2*, *CHEK2*, *PALB2*, *RAD51C*, *RAD51D,* and *BARD1* do not present specific phenotypes, but correlate with a higher risk in developing neoplasms. Alterations in these genes could induce the relative associated hereditary cancer syndrome. All these genes encode proteins involved in the maintenance of genome stability and DNA repair, mainly in the homologous recombination pathway for double-strand DNA repair [26,27,28,29]. These syndromes are mainly associated with an increased risk of developing breast and ovarian cancer [26,27,28,29,38]. 

In the *BRCA1*- and *BRCA2*-associated hereditary cancer syndromes, there is a higher risk of prostate cancer and gastrointestinal cancers (pancreatic, gastric, biliary tract, esophageal), while the risk of melanoma, colorectal, and lung cancer is still a matter for debate [26,27,28,29]. 

The *CHEK2*-associated hereditary cancer syndrome could include gastrointestinal tumors (colon and gastric cancer), prostate cancer, renal cancer, thyroid cancer, sarcomas, and hematological neoplasms (leukemia and plasma cell neoplasms) [28,38,47,48,49]. In this case, the phenotype is LS-like but more attenuated, and there is no strong evidence for associations as is the case for the mutation of *TP53*. It has been demonstrated that there is an association of germline CHEK2 variants with breast and prostate cancer but some of the data (VUS classification, patients’ geographic distribution, comprehensive CHEK2 mutation analysis) is heterogenous [70]. 

The *PALB2*-associated hereditary cancer syndrome is also associated with pancreatic cancer [28,29,33,34,35,36,37,38], while the *BARD1*-associated hereditary cancer syndrome is associated with colon cancer [28,38,44]. 

However, in the FA spectrum, unique phenotypes are associated with specific alterations in *BRCA2* (*FANCD1*), *PALB2* (*FANCN*), *RAD51* (*FANCR*), and *BRCA1* (*FANCS*) [56,57,58,59]. FA displays an autosomal recessive inheritance, but cancer risk is associated with some heterozygous mutations in the following genes: *BRCA2* (*FANCD1*), *BRIP1* (*FANCJ*), *PALB2* (*FANCN*), *RAD51C* (*FANCO*), *BRCA1* (*FANCS*) [71].

Hereditary ovarian cancer is associated with several genetic syndromes (Table 1, Table 2 and Table S1): *BRCA1*- and *BRCA2*-associated hereditary cancer syndrome (lft 17–44%; gene: *BRCA1*/*2*) [26,27,28,29]; *STK11*-associated hereditary cancer syndrome or Peutz–Jeghers syndrome (PJS) (up to 21%; gene: *STK11*) [30,31,32]; Lynch syndrome (LS) (lft 3–17%; gene: *MLH1, MSH6, MSH2, PMS2*) [31,64,72,73,74]; MAP (up to 14%; gene: *MUTYH*) [31,75]; *RAD51D*-associated hereditary cancer syndrome (up to 13%; gene: *RAD51D*) [28,38,52,53]; *RAD51C*-associated hereditary cancer syndrome (up to 11%; gene: *RAD51C*) [28,29,38,52]; *BRIP1*-associated hereditary cancer syndrome (up to 5.8%; gene: *BRIP1*) [28,76]; *PALB2*-associated hereditary cancer syndrome (up to 5%; gene: *PALB2*) [28,29,33,34,35,36,37,38]; ATM or AT or Louis-Bar syndrome (gene: *ATM*) [28,38,39,40]; PPAP (gene: *POLE*, *POLD1*) [60,61,62,63]; BSyn (gene: *BLM*) [67,77], DICER1 tumor predisposition (gene: *DICER1*) [78].

An overlap exists among the following hereditary syndromes associated with a higher risk of developing both breast and ovarian cancer: Ataxia telangiectasia [28,38,39,40], Bloom syndrome [67,77], Peutz–Jeghers syndrome [30,31,32], and Polymerase proofreading-associated polyposis [60,61,62,63].

As for the previous hereditary cancer syndromes related to breast cancer, there are some hereditary cancer syndromes related to ovarian cancer which are characterized by a typical phenotype: *DICER1* tumor predisposition, LS, and MAP-associated polyposis. 

*DICER1* tumor predisposition could present suggestive features such as macrocephaly, retinal abnormalities, multinodular goiter, renal alterations (cystic nephroma, abnormal of the collecting system of the kidney), nasal chondromesenchymal hamartoma, and dental anomalies. The hereditary syndrome is also associated with other neoplasms aside from ovarian sex cord–stromal tumors: pleuropulmonary blastoma, medulloepithelioma, differentiated thyroid carcinoma, embryonal rhabdomyosarcoma of the cervix, and central nervous system cancers (pituitary blastoma, pineoblastoma, sarcomas, presacral malignant teratoid tumor, other embryonal tumors). In this case, the mutated gene is *DICER1*, which encodes a double-stranded RNA endoribonuclease involved in gene suppression but which also acts as a strong antiviral agent against RNA viruses [78,79]. 

LS represents a well-known hereditary cancer syndrome mainly in gastrointestinal neoplasms and endometrial cancer, but it could also be responsible for an increased risk of developing ovarian cancer (endometrioid) as well as breast cancer, though less frequently. In addition, colon adenomas, mostly with high grade dysplasia and/or villous histology, are peculiar features of LS, similar to skin tumors (sebaceous neoplasms, keratoacanthomas). LS is caused by a mutation in at least one of the following genes: *MLH1 (MutL Homolog 1), MSH6 (MutS Homolog 6), MSH2 (MutS Homolog 2),* and *PMS2 (PMS1 Homolog 2)*. The encoded proteins are involved in DNA mismatch repair, creating MLH1–PMS2 and MSH2–MSH6 heterodimers. [31,64,72,73,74]. 

Among the hereditary cancer syndromes characterized by colonic polyposis, together with PJS and Polymerase proofreading-associated polyposis, MAP-associated polyposis is discussed below [31,75].

Considering hereditary ovarian cancer, as mentioned earlier, alterations in *BRCA1*/2, *CHEK2*, *PALB2*, *RAD51C*, *RAD51D*, and *BRIP-1* are associated with an increased risk of developing ovarian cancer, without a specific phenotype [26,27,28,29,33,34,35,36,37,38,47,48,49,76]. *BRIP-1*-associated hereditary cancer syndrome could also include a higher risk of developing breast cancer. The encoded protein BRIP-1 is involved in the DNA double-strand break repair by homologous recombination [28,76].

Considering the syndromes predisposing to prostate cancer, several overlaps with breast or ovarian cancer syndromes emerge (Table 1, Table 2 and Table S1): *BRCA1*- and *BRCA2*-associated hereditary cancer syndrome (gene: *BRCA1*/*2*) [26,27,28,29], ATM or AT or Louis-Bar syndrome (gene: *ATM*) [28,38,39,40], and Lynch syndrome (LS) (lft 5–16%).

*HOXB13*-hereditary cancer syndrome, associated with mutations in the *HOXB13* gene, is closely related to prostate carcinoma. *HOXB13*, a member of the homeobox family, codes for a protein with an important role in urogenital development and which retains high levels of prostate expression even in adults, although the molecular mechanism by which *HOXB13* promotes prostate cancer development remains unknown.

Similar to prostate cancer, pancreatic cancers are also associated with predisposing genetic syndromes in the mammaric/ovarian sphere: LF (gene: *TP53*) [24,25]; *BRCA1*- and *BRCA2*-associated hereditary cancer syndrome (lft 16%; gene: *BRCA1/2*,) [26,27,28,29]; *PALB2*-associated hereditary cancer syndrome (up to 3%; gene: *PALB2*) [28,29,33,34,35,36,37,38]; *ATM*-associated hereditary cancer syndrome or Ataxia telangiectasia (lft 9.5%; gene: *ATM*) [28,38,39,40]; *STK11*-associated hereditary cancer syndrome (up to 26%; gene *STK11*) or Peutz–Jeghers syndrome (up to 36%) [30,31,32].

Hereditary pancreatitis, on the other hand, is a syndrome predisposing specifically to pancreatic tumors. It is commonly characterized by an autosomal dominant inheritance pattern, typically associated in up to 80% of patients with mutations in the *PRSS1* gene, which encodes a cationic trypsin [80,81]. Clinically it presents with acute, acute recurrent, and chronic pancreatitis, and it is one of the main causes of pancreatitis in childhood. The syndrome can also be attributed to less frequently occurring genes, namely, *SPINK I*, *CFTR*, and *CTRC*. *SPINK I* encodes an acute-phase protein that functions as a trypsin inhibitor, preventing autodigestion of the pancreatic parenchyma by inhibiting the activation of digestive enzymes. The *CFTR* mutation is associated with several symptoms, including sinusitis, respiratory distress, male infertility, and constipation. *CTRC*, on the other hand, encodes a protease that can degrade trypsin and trypsinogen. 

While less common, it is noteworthy that the FAMM syndrome, which has already been extensively discussed in the melanoma section, can predispose up to 17% of patients to pancreatic cancer.

## 6. HCS of Neuroendocrine Tumors

Neuroendocrine tumors are linked to several genetic syndromes (Table 1, Table 2 and Table S1), including Multiple endocrine neoplasia type 1 (MEN1), type 4 (MEN4), and von Hippel–Lindau syndrome. 

MEN1, an autosomal dominant disorder, is caused by germline mutations in the *MEN1* tumor suppressor gene that encodes menin [82,83], a ubiquitously expressed protein with numerous functions such as transcriptional regulation, genomic stability, cell division, and proliferation [84,85,86]. In MEN1, parathyroid tumors are the most frequent neuroendocrine tumors, observed in over 85% of patients, leading to primary hyperparathyroidism [87,88]. MEN1 is also associated with gastrinomas, insulinomas, glucagonomas, VIPomas, and somatostatinomas. Up to 33% of MEN1 patients may develop breast carcinoma, and up to 25% may develop carcinoids (thymic, bronchial, Type II gastric enterochromaffin-like carcinoid). Less commonly associated neoplasms include adrenocortical carcinomas (cortisol-secreting, aldosterone-secreting), pheochromocytomas, and thyroid carcinomas. Additionally, benign/preneoplastic lesions such as parathyroid adenomas; pituitary adenomas (prolactinomas, growth hormone, rare TSH, ACTH, nonfunctioning); adrenocortical tumors such as cortical adenomas or hyperplasia; skin findings, including facial angiofibromas, collagenomas, and lipomas; meningiomas and ependymomas in the central nervous system; and thyroid adenomas are associated with MEN1.

MEN4 is a recently discovered syndrome caused by germline mutations in the tumor suppressor gene *CDKN1B*, which codes for the cell cycle-regulating protein p27 that inhibits cyclin/cyclin-dependent kinase complexes. While the malignant component is unknown, approximately 25% of MEN4 patients may develop neuroendocrine tumors, including gastrinomas potentially leading to peptic and gastric ulcerations, insulinomas, glucagonomas, VIPomas, and somatostatinomas. MEN4 is also linked with carcinoids and adrenocortical carcinomas secreting cortisol or aldosterone. Up to 80% of MEN1 patients develop primary hyperparathyroidism associated with parathyroid adenomas and with a female predominance [89], while up to 25% develop pituitary adenomas (prolactinomas, growth hormone, rare TSH, ACTH, nonfunctioning).

The von Hippel–Lindau syndrome, as discussed in the section on renal tumors, is an autosomal dominant disorder that predisposes individuals to a range of malignant and benign tumors, including renal cell carcinoma (clear cell subtype), pheochromocytoma, CNS hemangioblastomas, and retinal angiomas. Up to 12% of patients with vHL syndrome may develop pancreatic neuroendocrine tumors (PNETs), although most pancreatic lesions associated with the vHL syndrome are cysts or serous cystadenomas [90]. In the case series of Blansfield et al., 8.3% had metastatic disease from their PNETs, and lesions larger than 3 cm were more likely to develop metastases (*p* < 0.005) [90].

## 7. HCS of Esophageal and Gastric Cancer

Gastric and esophageal cancers display a peculiar geographic, ethnic, and socioeconomic distribution, with higher incidence in Asian and developing countries [91]. Well-established predisposing conditions consist of environmental and dietary factors, including H. pylori infection, alcohol consumption, and tobacco smoking, with a synergistic effect, along with high intake of processed meat [92]. Most cases are sporadic, and familiar aggregation is reported in nearly 10% of patients, while less than three percent of cancer cases is attributable to hereditary syndromes [93]. 

With regard to gastric cancer (GC), the hereditary component is mainly related to two major syndromes: hereditary diffuse gastric cancer (HDGC) and gastric adenocarcinoma and proximal polyposis of the stomach (GAPPS), with different pathogenetic mechanisms. 

HGDC is caused by germline mutations in the cadherin 1 (*CDH1*) gene, which encodes the cell adhesion protein E-cadherin [69]. It is a rare, highly penetrant, autosomal dominant disease characterized by higher susceptibility to developing diffuse and poorly differentiated gastric adenocarcinoma (DGC) infiltrating the stomach wall (*linitis plastic*) without forming a mass [43]. The cumulative risk of GC by the age of 80 years is estimated to be up to 70% for males and 56% for females, with a median age at GC diagnosis of 38 years within a wide range from 14 to 69 years [41]. Lobular breast cancer (LBC) and cleft lip/palate are also distinctive features of syndromic patients [42]. Women with a pathogenetic variant, in fact, carry a 42% lifetime risk of lobular breast cancer [41]. According to the 2020 International Gastric Cancer Linkage Consortium (IGCLC) consensus guidelines, HDGC should be suspected and *CDH1* testing should be offered in the presence of [41] of certain family criteria (≥2 cases of GC in family regardless of age, with at least one DGC; ≥1 case of DGC at any age, and ≥1 case of LBC at <70 years in different family members; ≥2 cases of LBC in family members <50 years) or individual criteria (DGC at <50 years; DGC at any age in the case of Māori ethnicity; DGC at any age in individuals with a personal or family history (first-degree relative) of cleft lip/palate; history of DGC and lobular breast cancer, both diagnosed at <70 years; bilateral LBC diagnosed at <70 years; gastric in situ signet ring cells or pagetoid spread of signet ring cells diagnosed at <50 years).

In the spectrum of the polyposis syndromes of the GI tract, gastric adenocarcinoma and proximal polyposis of the stomach (GAPPS) represents a rare, autosomal dominant with incomplete penetrance variant of familial adenomatous polyposis (FAP) [94]. 

GAPPS is caused by specific point mutations in promoter 1B regions of the *APC* gene that reduce gastric *APC* expression [95]. The *APC* gene is an oncosuppressor that encodes a protein involved in the control of cell adhesion though the negative regulation of β-catenin and E-cadherin interaction, thus preventing uncontrolled cell growth and cell migration [96]. Typical endoscopic findings include more than 100 gland polyps in the gastric body and fundus with antral sparing. Patients display increased susceptibility to intestinal gastric type adenocarcinoma due to malignant polyp degeneration, with a lifetime risk of 13–25% [97,98,99]. The GAPPS phenotype differs from that of FAP as polyposis is restricted to the proximal stomach without involvement of the large intestine [94]. Nevertheless, FAP patients can have gastric polyps, and, in rare cases, gastric cancer develops from a dysplasia–carcinoma pathway as an extracolonic manifestation of the FAP syndrome [100,101]. Molecular genetic testing for GAPPS should be considered in individuals with gastric polyps restricted to the body/fundus, more than 100 polyps in the index case or more than 30 polyps in a first-degree relative, fundic gland polyp histology, autosomal dominant pattern of inheritance, and exclusion of other gastric polyposis syndromes. 

Gastric cancer is also associated with a wide range of several cancer-associated syndromes [97]. LS is one of the most common inherited GI cancer syndromes and, according to the latest estimates, the lifetime risk of developing gastric cancer in these patients ranges from 1% to 13% [102]. In contrast to FAP and GAPPS, LS patients, despite a similar histology of GI neoplasms, do not present with polyposis. Moreover, in contrast with HDGC, LS-associated gastric cancers display intestinal histology. 

A large case series also described a rate of approximately 5% of GC cases with both diffuse and intestinal-type histology in LF patients [103]; of these, there was a 29% cumulative risk of gastric cancer up to age 64 years in patients with germline mutations of *STK11* [104]; a lifetime GC risk of 21% in individuals with Juvenile Polyposis syndrome [105] and approximately 20% in *BRCA1/2* mutation carriers [106]. 

Extremely rare genetic conditions are linked to esophageal cancers (EC). In particular, the Howel–Evans syndrome is characterized by distinctive cutaneous features such as hyperkeratosis of the palms of the hands and soles of the feet (tylosis), and it is strongly associated with esophageal squamous cancer, favored by traditional risk factors [107]. 

Moreover, hereditary deleterious variations of *BRCA2*, *ATM, STK11, FA*, and *FAMM* may confer an increased risk of EC.

## 8. HCS of Colorectal Cancer

Genetic predisposition due to the presence of germline pathogenetic mutations has been implicated in approximately 5–6% of colorectal cancer cases (CRCs) [108]. According to current guidelines, screening for hereditary CRC syndromes should include a review of personal and family cancer history, universal testing for MMR status, and individuation of suggestive syndromic clinical features [108]. Genetic evaluation of suspected cases is highly recommended in the case of suggestive factors such as a strong family history for CRC and/or polyps, CRC diagnosis at early age, and multiple primary cancers in a CRC patient [109]. In particular, hereditary syndromes can be divided into two main categories depending on the presence or absence of polyp formation. 

Among polyposis syndromes, the most frequent are familial adenomatous polyposis (FAP) and its variants, such as the Gardner syndrome and attenuated familial polyposis syndrome (AFAP), which are all caused by germline mutations in the *APC* tumor suppressor gene with an autosomal dominant pattern of inheritance [94]. 

Classic FAP is characterized by the presence of more than 100 colorectal adenomas, with a lifetime risk of CRC of >90% and a median age of CRC onset in untreated individuals of 39 years. It is responsible for ≤1% of all CRC diagnoses [110]. Extracolonic manifestations, so-called the Gardner syndrome, are frequent and include gastric fundic gland polyps, duodenal adenomas, nasopharyngeal angiofibromas, osteomas, fibromas, dental abnormalities, thyroid nodules, epidermoid or sebaceous cysts, and congenital hypertrophy of the retinal pigment epithelium (CHRPE) [18]. *APC*-mutation carriers also display a higher risk of duodenal and periampullary cancer, brain tumors (Turcot syndrome), thyroid papillary carcinoma, hepatoblastoma, pancreatic cancer, and desmoid tumors [111]. 

Attenuated familial adenomatous polyposis (AFAP) is a phenotypic variant of FAP and is characterized by the presence of 10–99 colonic adenomas, delayed polyp development, and subsequently older age at cancer diagnosis compared with the classic form [112]. In AFAP, the magnitude of the CRC risk is strictly dependent on the severity of the phenotype. Despite similar clinical features, attenuated and classic polyposis syndromes differ in the number and the timing of polyp formation, and, as direct consequence, the age of CRC diagnosis. Moreover, extracolonic manifestations are extremely rare in attenuated forms [112]. 

An *APC*-associated polyposis condition should be suspected if any of the following criteria is present: at least 10–20 cumulative colorectal adenomatous polyps, a family history of multiple colorectal polyps, and having a known *APC* variant and/or typical extracolonic features [113]. 

Other less frequent polyposis syndromes include rare entities which represent a clinical challenge in terms of diagnosis and management as consequence of their low incidence and some overlapping features [114]. MAP is an autosomal recessive disorder caused by pathogenetic variants in *MUTYH*, which encodes a DNA glycosylase belonging to the base excision repair complex [115]. The MUTYH protein, in fact, plays a role in the repair of single-stranded breaks following oxidative damage [75]. The clinical characteristics resemble those FAP or AFAP and include from 10 to >100 colorectal polyps with an increased risk of developing CRC at a median age of 50 years [116]. Of note, patients carrying heterozygosis *MUTYH* alterations can develop CRC even without polyposis [117]. The frequency of MUTYH heterozygotes in the general population is approximately 2% [118]. Large studies found that individuals with monoallelic MUTYH alterations display twice the population cancer risk of CRC [119]. However, current evidence regarding CRC risk is still lacking, and no specific guidelines have been developed. Other features are not universally accepted (benign adrenal lesions, thyroid nodules, skeletal alterations (jawbone cysts, osteomas), congenital hypertrophy of the retinal pigment epithelium (CHRPE), desmoid tumors, and epidermoid or sebaceous cysts).

Current multigenic panels also include *MSH3* and *MLH3*, mismatch repair genes not associated with LS. The presence of biallelic variants is implicated in polyposis syndromes with an AFAP-like phenotype (adenomatous polyposis at early age and extracolonic malignant and benign lesions) called *MSH3/MLH3*-associated polyposis [68,120]. 

PJS and Juvenile Polyposis syndrome (JPS) are characterized by an increased CRC risk caused by the presence of hamartomatous gastrointestinal polyps. Both syndromes are also associated with extracolonic cancers: breast, pancreatic, and gynecological cancers for PJS and upper GI cancers for JPS [121]. 

Colonic serrated polyps are typical of sessile serrated polyposis cancer syndrome (SSPCS), with an overall CRC risk of approximately 20% [122]. 

In the *NTHL1* tumor syndrome (NTHL1), polyps’ histology may vary with adenomatous, hyperplastic, or serrated polyps. The *NTHL1* gene encodes a protein that excises DNA base damage caused by reactive oxygen species [123]. The CRC risk is increased, and carriers also display susceptibility to a wide range of other solid tumors, such as gynecological, urothelial, and brain cancer, basal cell carcinomas, head and neck squamous cell carcinomas, and hematologic malignancies [124]. 

Other rare causes of inherited polyposis include the PPAP syndrome or hereditary mixed polyposis [125].

Among nonpolyposis syndromes, the Lynch syndrome has a central role. LS is responsible for nearly 3% of all colon cancers and is associated with a germline mutation in the mismatch repair (MMR) genes (*MLH1, MSH2, MSH6,* and *PMS2*), leading to the loss of expression of their respective proteins [126]. The DNA MMR is a strand-specific system involved in recognizing and repairing erroneous replicative pairings during DNA synthesis [127]. As result, LS carriers have impaired MMR, a hypermutable status, and microsatellite instability [128]. This autosomal dominant syndrome is considered the most frequent genetic cause of CRC; therefore, current guidelines recommend universal screening for LS with immunohistochemical techniques [129]. The proteins encoded by MMR genes form heterodimers: MLH1 builds a functional complex with PMS2, and MSH2 dimerizes with MSH6. MLH1 and MSH2 are called obligatory proteins, while PMS2 and MSH6 are considered secondary partners [127]. Therefore, mutations in MLH1 or MSH2 lead to the degradation of the dimer and subsequent loss of both respective proteins. When MMR status is assessed in IHC, if there is a loss of MLH1 protein expression, a concurrent loss of PMS2 protein expression is generally also observed [130]. Similarly, the same phenomenon occurs for MSH2 and MSH6. However, the loss of secondary partners (PMS2 or MSH6) does not necessarily imply the loss of obligatory partners. Germline deletions of the EPCAM gene can also lead to epigenetic silencing of MSH2, thus causing Lynch syndrome [131]. LS patients are mainly at risk of developing colorectal and endometrial cancers, with an estimated lft ranging from 30 to 70% [126]; less frequently, carriers can present with gastric, pancreatic, biliary, ovarian, and urinary tract cancers [132,133]. Diagnostic criteria for LS based on personal and family history include the Amsterdam II criteria and the revised Bethesda guidelines [134]; however, some studies reported a lack of testing sensitivity, so in any case of clinical suspicion, a patient should be referred for genetic evaluation [135].

*RPS20*-associated hereditary nonpolyposis is related to an increased CRC risk. This is a rare, autosomal dominant disease that differs from LS by the absence of microsatellite instability and no susceptibility to malignancies other than CRC [136].

## 9. Conclusions

The heterogeneity and growing number of genetic syndromes that underlie oncological pathologies necessitate a global comprehension of their particular clinical manifestations. Recently, advances in molecular analysis technologies have facilitated the availability of increasingly sophisticated genomic panels, which significantly enhance diagnostic capabilities. This has brought to light even more the importance of accurate differential diagnoses, which provide primary care physicians with the necessary tools for the early detection of suspicious patients. Early clinical diagnoses are indeed beneficial in several ways: they not only facilitate the identification of patients at risk of specific medical complications and prompt the application of appropriate treatment, but they also allow for improved counseling and risk/benefit analysis, positively impacting the psychological well-being of both patients and their families.

From a clinical perspective, it is of particular interest to observe that a single pathogenic gene variant can cause a heterogenous phenotype while, on the other hand, the same phenotype can be caused by different gene pathogenic variants. This complex scenario strengthens the necessity of developing validated predictive tools; for example, through machine learning. However, clinical knowledge of genotype–phenotype correlations still remains a pivotal skill for modern physicians. Moreover, the extreme number of variables highlights the need for a multidisciplinary team for the proper management of HCS.

A limitation of our work is the paucity of data for some syndromes and the weak or hypothetical sociation of some phenotypes with some HCSs. However, there are ongoing international studies that aim to level these gaps of knowledge (e.g., ClinGen, https://clinicalgenome.org/, accessed on 22 March 2023).

Accordingly, the study of genetic syndromes remains a rapidly evolving field. On the one hand, the ongoing efforts towards massive genomic sequencing, driven by significant financial and technological investments, are likely to lead to the discovery of new, previously unknown rare syndromes in the coming years. On the other hand, the clinical application of polygenic risk scores (PRS)—which quantify an individual’s risk of developing a particular disease through the evaluation of a score derived from single nucleotide polymorphisms present in a genome—will allow for personalized risk assessments, which are beneficial for prevention, early diagnosis, and disease management. Juxtaposing clinical knowledge with technological advances is and will be indispensable to best manage the complexity of hereditary cancer syndromes.

## Figures and Tables

**Table 1:** A comprehensive list of hereditary cancer syndromes. AD = autosomal dominant; AR = autosomal recessive; M = mixed; U = unknown; X = X-linked. Sources: Pubmed, Web of Science, GeneReview, UptoDate, Omim, EviQ Genetics.

Syndrome	Acronym	Prevalence	Inheritance	Involved Genes
Hereditary paraganglioma-pheochromocytoma syndrome	HPPS	1–9:1,000,000	AD (SDHA, SDHB, SDHC, TMEM127) Paternal inheritance (SDHD, SDHAF2, MAX)	*SDHA, SDHAF2, SDHB, SDHC, SDHD, MAX, TMEM127*
Carney Complex	CNC	U	AD	*PRKAR1A*
Neurofibromatosis type 1	NF1	1:2600	AD	*NF1*
Neurofibromatosis type 2	NF2	1:60,000	AD	*NF2*
Schwannomatosis	SCHW	1:70,000	AD	*SMARCB1, LZTR1*
Multiple endocrine neoplasia type 1	MEN 1	1:10,000	AD	*MEN1*
Multiple endocrine neoplasia type 2A	MEN2A	1:44,000	AD	*RET*
Multiple endocrine neoplasia type 2B	MEN2B	1:700,000	AD	*RET*
Familial medullary thyroid carcinoma	FMTC	1:233,000	AD	*RET*
Multiple endocrine neoplasia type 4	MEN4	<1:1,000,000	AD	*CDKN1B*
Hyperparathyroidism-jaw tumor syndrome	HPT-JT	U	AD	*CDC73*
Parathyroid carcinoma syndrome	PC	U	AD	*CDC73*
HOXB13 hereditary cancer syndrome	HOXB13	U	AD	*HOXB13 G84E*
NBN hereditary cancer syndrome	NBN	U	AD (heterozygous)	*NBN*
Njimegen Breakage syndrome	NBS	1:100,000	AR (homozygous)	*NBN*
Von Hippel–Lindau syndrome	VHLS	1:36,000	AD	*VHL*
Hereditary papillary renal carcinoma syndrome	HPRC	U	AD	*HPRC (MET* protooncogene)
Hereditary leiomyomatosis and renal cancer cell syndrome	HLRCC	<1:500	AD	*FH*
Tuberous Sclerosis Complex	TSC	1:5800	AD	*TSC1, TSC2*
Birt–Hogg–Dubé syndrome	BHD	1:500,000	AD	*FLCN*
Li–Fraumeni syndrome	LF	1:3500	AD	*TP53*
Bloom syndrome	BSyn	U	AR	*BLM*
Familial GIST	fGIST	U	AD	*KIT, PDGFRA*
BRCA1- and BRCA2-associated hereditary cancer syndrome	BRCA1/2	1:500 (BRCA1) 1:225 (BRCA2)	AD	*BRCA1, BRCA2*
CHEK2-associated hereditary cancer syndrome	CHEK2	1:937 (CHEK2 R95)	AD	*CHEK2*
PALB2-associated hereditary cancer syndrome	PALB2	1:1250	AD	*PALB2*
RAD51C-associated hereditary cancer syndrome	RAD51C	1:1600	AD	*RAD51C*
RAD51D-associated hereditary cancer syndrome	RAD51D	U	AD	*RAD51D*
ATM-associated hereditary cancer syndrome	ATM (heterozygosis)	1:100	AR	*ATM*
Ataxia telangiectasia	ATM (homozygosis)	1:40,000–300,000	AR	*ATM*
Peutz–Jeghers syndrome	STK11	1:25,000–280,000	AD	*STK11*
BARD1-associated hereditary cancer syndrome	BARD1	U	AD	*BARD1*
BRIP1-associated hereditary cancer syndrome	BRIP1	1:500	AD	*BRIP1*
Fanconi Anemia	FA	1–9:1,000,000 The carrier frequency of FA is 1/181 in the general population in North America and 1:93 in Israel. Specific populations have a founder effect with increased carrier frequencies (1 per 100 or less)	M (AR, X)	*AR: FANCA, FANCC, FANCD1/BRCA2, FANCD2, FANCE, FANCF, FANCG (XRCC9), FANCI, FANCJ/BRIP1, FANCL, FANCM, FANCN/PALB2, FANCP/SLX4, FANCQ/ERCC4, FANCR/RAD51, FANCS/BRCA1, FANCT/UBE2T, FANCU/XRCC2, FANCV/REV7, FANCW/RFWD3, and FANCY/FAP100* *X*: a hemizygous pathogenic variant in *FANCB*
Familial atypical mole-malignant melanoma syndrome	FAMM	U	AD	*CDKN2A, CDK4*
Nevoid basal cell carcinoma	NBCC	1:31,000–164,000	AD	*PTCH1, SUFU*
Xenoderma pigmentosus	XP	1:1,000,000 (EU, USA), 1:22,000 (JAP)	AR	*DDB2 (XPE), ERCC1, ERCC2 (XPD), ERCC3 (XPB), ERCC4 (XPF), ERCC5 (XPG), POLH (XPV), XPA, XPC*
BAP1 tumor predisposition syndrome	BAP1	U	AD	*BAP1*
Shelterin complex genes hereditary cancer syndrome	Shelterin	U	AD	*POT1, ACD, TERF2IP*
TERT hereditary cancer syndrome	TERT	U	AD/AR	*TERT*
MC1R polymorphism	MC1R	U	AD	*MC1R*
MITF (E318K) polymorphism	MITF	U	AD	*MITF*
DICER1 tumor predisposition syndrome	DICER1	U	AD	*DICER1*
Lynch syndrome	LS	1:279	AD	*MLH1, MSH2, MSH6, PMS2, EPCAM*
RPS20-associated hereditary nonpolyposis colorectal cancer syndrome	RPS20	U	AD	*RPS20*
Familial adenomatous polyposis	FAP	1:8000	AD	*APC*
Attenuated familial adenomatous polyposis	AFAP	U	AD	*APC*
Gastric adenocarcinoma and proximal polyposis of the stomach	GAPPS	U	AD	*APC*
Polymerase proofreading-associated polyposis	PPAP	U	AD	*POLE, POLD1*
MUTYH-associated polyposis	MAP	1:20,000 (carrier 1:100)	AR	*MUTYH*
NTHL1 tumor syndrome	NTHL1	U	AR	*NTHL1*
MSH3-associated polyposis	MSH3	<1:1,000,000	AR	*MSH3*
MLH3-associated polyposis	MLH3	U	AR	*MLH3*
Juvenile polyposis syndrome	JPS	U	AD	*BMPR1A, SMAD4*
Hereditary mixed polyposis syndrome	HMPS	U	AD	*GREM1*
Sessile serrated polyposis cancer syndrome	RNF43	U	AD	*RNF43*
Cowden syndrome	CS	1:200,000	AD	*PTEN*
Hereditary diffuse gastric cancer syndrome	HDGC	U	AD	*CDH1*
Hereditary pancreatitis	HP	1–9:1,000,000	AD (PRSS1, CFTR, SPINK1, CPA1, CTRC, CASR, CEL) X (CLDN2) AR (CFTR, SPINK1, TRPV6)	*PRSS1, SPINK1, CFTR, CTRC, CLDN2, CPA1* (Putative genes: *CEL, CELP, CASR, GGT1, TRPV6*)
Howel–Evans syndrome	HE	<1:1,000,000	AD	*RHBDF2*
EGFR-associated genetic susceptibility	EGFR	U	AD	*EGFR*

**Table 2:** List of malign and benign features of hereditary cancer syndromes. HCS = hereditary cancer syndrome. Blue boxes identify a typical features of that syndrome; black box identify features that are commonly associated with that syndrome; gray box identify features that rarely associated with that syndromes or the association is weakly supported by the literature. The readers can find the benign/malign phenotype of a patient/family in the table to better hypothesize the underlying HCS. Sources: GeneReview, UptoDate, Omim, EviQ Genetics.

	HPPS	CNC	NF1	NF2	SCHW	MEN 1	MEN2A	MEN2B	FMTC	MEN4	HPT- JT	PC	HOXB13	NBN	NBS	VHLS	HPRC	HLRCC	TSC	BHD	LF	BSyn	fGIST	BRCA1/2	CHEK2	PALB2	RAD51C	RAD51D	ATM (Heterozygosis)	AT	STK11	BARD1	BRIP1	FANCONI ANEMIA	FAMM	NBCC	XP	BAP1	Shelterin	TERT	MC1R	MITF	DICER1	LS	RPS20	FAP	AFAP	GAPPS	PPAP	MAP	NTHL1	MSH3	MLH3	JPS	HMPS	RNF43	CS	HDGC	HP	HE	EGFR
HCS-associated cancers																																																													
Esophagus/Stomach																																																													
Small bowel																																																													
Colon/Rectum																																																													
Anus																																																													
Pancreas																																																													
Liver																																																													
Biliary tract																																																													
Breast																																																													
Ovary																																																													
Endometrium																																																													
Cervix																																																													
Vulva/Vagina																																																													
Prostate																																																													
Testicles																																																													
Penis																																																													
Kidney																																																													
Bladder/Urothelial																																																													
Melanoma																																																													
Skin (non-melanoma)																																																													
Lung																																																													
Mesothelium																																																													
Head & neck																																																													
Sarcoma																																																													
GIST																																																													
Neuroendocrine																																																													
Pheocromocytoma/Paraganglioma																																																													
Adrenal																																																													
Thyroid																																																													
Parathyroid																																																													
Nervous system																																																													
Lymphoma																																																													
Leukemia																																																													
Thymus																																																													
Parotid																																																													
Endolymphatic sac tumors																																																													
Choriocarcinoma																																																													
HCS-associated benign features																																																													
Eyes																																																													
Nose																																																													
Mouth																																																													
Esophagus/Stomach																																																													
Small bowel																																																													
Colon/Rectum																																																													
Biliary tract																																																													
Pancreas																																																													
Kidney																																																													
Bladder/Urothelium																																																													
Breast																																																													
Testicular																																																													
Gynecological																																																													
Skin/Soft tissue																																																													
Lung																																																													
Skeleton																																																													
Adrenal																																																													
Thyroid																																																													
Parathyroid adenoma																																																													
Pituitary gland																																																													
Nervous system																																																													
Cognitive alterations																																																													
Heart																																																													
Facies/Habitus																																																													
Blood																																																													
Blood vessels																																																													
Thymus																																																													
Parotid																																																													
Liver																																																													
Immune system																																																													
Endocrine dysfunction																																																													

## Supplementary Materials

The following supporting information can be downloaded at: https://www.mdpi.com/article/10.3390/genes14051025/s1, Table S1: Detailed features of hereditary cancer syndromes with the lifetime risk of various cancers associated with each syndrome.
