# Supplementary material for: Hereditary Cancer Syndromes: A Comprehensive Review with a Visual Tool
Source: Genes (Basel). 2023 Apr 30;14(5):1025. doi: 10.3390/genes14051025 (PMC10218093; doi:10.3390/genes14051025)
Supplement: Supplementary file 1 [file genes-14-01025-s001.zip › genes-2336563-Supplementary.pdf]

Syndrome	Cancers	Benign or preneoplastic alterations
Hereditary paraganglioma-pheochromocytoma syndrome (HPPS)	Paragangliomas and/or Pheochromocytomas (up to 41%)<br>Gastrointestinal stromal tumors (GISTs) (2-more than 5%; GIST are more common than paragangliomas/pheochromocytomas in SDHA pathogenetic variant)<br>Renal clear cell carcinoma (2-5%)<br>Papillary thyroid carcinoma **(R)**<br>Neuroendocrine tumors **(R)**	Paragangliomas and/or Pheochromocytomas (up to 64%)<br>Pulmonary chondromas<br>Adrenal cortical adenoma<br>Esophageal leiomyoma<br>Pituitary adenomas **(R)**
Carney Complex (CNC)	Thyroid carcinoma (papillary, follicular)(typically at young age)<br>Psammomatous melanotic schwannoma (PMS)<br>Ovarian endometrioid carcinoma **(R)**<br>Testicular cancer **(R)**	Skin pigment abnormalities:<br>- Pale brown to black lentigines on skin and mucosa (typical on lips, conjunctiva and inner or outer canthi, vaginal and penile mucosa)<br>- Epithelioid-type blue nevi<br>- Combined nevi<br>- Café au lait macules<br>- Depigmented lesions<br>Myxomas:<br>- Myxomas of skin, cardiac, breast, oropharynx, cervix, uterus, vagina<br>- Osteochondromyxoma (nasal sinuses and long bones)<br>Endocrine dysregulation:<br>- Primary pigmented nodular adrenocortical disease (PPNAD)(causing Cushing syndrome)<br>- Growth hormone (GH)-producing pituitary adenoma<br>- Testicular tumors (Large-cell calcifying Sertoli cell tumors (LCCSCT), Leyding cell tumors, nodular adrenocortical rest)(may produce hormones)<br>- Thyroid follicular adenomas (often nonfunctioning)<br>Psammomatous melanotic schwannoma (PMS)<br>Breast ductal adenoma<br>Ovarian cysts, serous cystadenoma, teratomas
Neurofibromatosis type 1 (NF1)	Malignant peripheral nerve sheath tumors (MPNSTs) and/or Gliomas (optic nerve, brain/spinal cord) (33%)<br>Gastrointestinal Stromal Tumors (GISTs) (6%)<br>Pheochromocytoma and/or paragangliomas (0.24%)<br>Rhabdomyosarcoma (RMS)<br>Breast Cancer (often estrogen receptor negative and human epidermal growth factor receptor 2 (HER-2) positive)<br>Juvenile myelomonocytic leukemia **(R)**	Cutaneous Features:<br>- Café au lait macules<br>- Intertriginous freckling (e.g., axillary, inguinal)<br>- Plexiform/Nodular neurofibromas<br>- Juvenile xanthogranuloma<br>- Nevus anemicus<br><br>Ocular findings:

	Neuroendocrine tumors **(R)**<br>Melanoma **(R)**<br>Ovarian cancer **(R)**<br>Hodgkin lymphoma **(R)**	- <u>Optic Pathway Gliomas (OPGs)</u><br>- <u>Lisch nodules (iris hamartomas)</u><br>- Choroidal freckling<br>- Retinal vasoproliferative tumors **(R)**<br>- Neovascular glaucoma **(R)**<br><br>Neurologic manifestations:<br>- Cognitive deficits<br>- Learning disabilities<br>- Sleep disturbance<br>- Autism spectrum disorders<br>- Seizures (usually not attributable to a mass lesion in the brain)<br>- Macrocephaly<br>- Peripheral neuropathy<br>- Multiple nerve root tumors<br>- Low-grade astrocytomas<br>- Brainstem gliomas<br>- Cerebellar gliomas<br>- Glomus Tumors<br><br>Musculoskeletal Features:<br>- <u>Sphenoid wing dysplasia</u><br>- <u>Long bones dysplasia (tibia and fibula)</u><br>- <u>Pseudoarthrosis</u><br>- Short stature<br>- Scoliosis (dystrophic or nondystrophic type)<br>- Nonossifying fibromas<br>- Vertebral dysplasia<br>- Reduced muscle strength<br>- Osteopenia<br><br>Vascular involvement:<br>- Essential Hypertension<br>- Vasculopathy (renal artery stenosis, coarctation of the aorta, Stenotic/ectatic cerebral arteries, cerebrovascular abnormalities)<br><br>Cardiac Issues:

		- Valvular pulmonic stenosis<br>- Congenital heart defects<br>- Hypertrophic cardiomyopathy<br>- Pulmonary Hypertension (often in association with parenchymal lung disease)<br><br>Pheochromocytomas and/or paragangliomas (1.76%)<br>Irritable bowel syndrome
Neurofibromatosis type 2 (NF2)	Ependymomas and gliomas **(R)**<br>Malignant peripheral nerve sheath tumors (MPNSTs) **(R)**	<u>Vestibular schwannomas (bilateral)</u><br><u>Ependymomas</u><br><u>Cranial, spinal, and peripheral nerves schwannomas</u><br><u>Meningiomas (most intracranial, but also intradural, extramedullary spinal meningiomas)</u><br>Neuropathies:<br>- Mononeuropathy (facial nerve)<br>- Polyneuropathy<br>Ophthalmic manifestations:<br>- <u>Cataracts</u><br>- Optic nerve meningiomas<br>- Retinal hamartomas<br>- Epiretinal membrane<br>Cutaneous manifestations:<br>- Plaque-like lesions<br>- Subcutaneous nodules<br>- Intracutaneous tumors (mostly schwannomas, but also neurofibromas)
Schwannomatosis (SCHW)	Schwannomas **(R)** *(malignant transformation of schwannomas remains a theoretic risk)*<br>Malignant peripheral nerve sheath tumors (MPNSTs) **(R)**<br>Rhabdoid tumors **(R)**	<u>Schwannomas of the peripheral/spinal/subcutaneous nerves</u><br>Schwannomas of the cranial nerves (trigeminal nerve) **(R)**<br>Unilateral vestibular schwannomas (bilateral vestibular schwannoma is an exclusion criterion)<br>Meningiomas (SMARCB1 variants)<br>Pain (in the majority of patients, pain does not correlate with a discrete mass)
Multiple endocrine neoplasia type 1 (MEN1)	<u>Neuroendocrine tumors (gastrinoma, insulinoma, glucagonoma, VIPoma, somatostatinoma, nonfunctioning)</u> (90%)<br>Breast cancer (33%)<br>Carcinoid (thymic, bronchial, Type II gastric enterochromaffin-like carcinoid) (25%)	<u>Parathyroid adenoma (hormone secreting)</u><br><br><u>Pituitary adenomas (prolactinomas, growth hormone, TSH rare, ACTH, nonfunctioning)</u>

	Adrenocortical carcinomas (cortisol-secreting, aldosterone-secreting) **(R)**<br>Pheochromocytoma **(R)**<br>Thyroid carcinomas **(R)**	Adrenocortical Tumors:<br>- Cortical adenomas<br>- Cortical hyperplasia<br>- Multiple adenomas<br>- Nodular hyperplasia<br>- Cysts<br>- Pheochromocytoma **(R)**<br><br>Skin findings:<br>- Facial angiofibromas<br>- Collagenomas<br>- Lipomas<br>- Café au lait macules<br>- Confetti-like hypopigmented macules<br>- Multiple gingival papules<br><br>Central nervous system tumors:<br>- Meningioma<br>- Ependymoma<br><br>Leiomyomas<br>Thyroid adenomas, thyroid colloid goiters
Multiple Endocrine Neoplasia type 2 (MEN2A)	<u>Medullary carcinoma of the thyroid (MTC)</u> (up to 90%)<br><u>Pheochromocytoma</u> **(R)**	<u>Parathyroid adenoma/ hyperplasia</u><br>C-cell hyperplasia (CCH)<br><u>Pheochromocytoma</u> (upto to 88%)<br>Cutaneous lichen amyloidosis **(R)**<br>Hirschsprung disease (HSCR)
Multiple Endocrine Neoplasia type 2 (MEN2B)	<u>Medullary carcinoma of the thyroid (MTC)</u> (up to 100%)<br><u>Pheochromocytoma</u> **(R)**	C-cell hyperplasia (CCH)<br><u>Pheochromocytoma</u> (up to 50%)<br>Mucosal neuromas (lips, tongue, pharynx, palate)<br>Submucosal nodules (lips)<br>Distinctive facies with enlarged lips<br>Ganglioneuromatosis of the gastrointestinal tract<br>Marfanoid habitus
Familial medullary thyroid carcinoma (FMTC)	<u>Medullary carcinoma of the thyroid (MTC)</u>	C-cell hyperplasia (CCH)<br>Hirschsprung disease (HSCR)
Multiple Endocrine Neoplasia type 4 (MEN4)	<u>Neuroendocrine tumors (gastrinoma, insulinoma, glucagonoma, VIPoma, somatostatinoma, nonfunctioning)</u> (25%, unknown the malignant component)	<u>Parathyroid adenoma (hormone secreting)</u>

	Carcinoid<br>Adrenocortical carcinomas (cortisol-secreting, aldosterone-secreting) **(R)**<br>Testicular cancer **(R)**<br>Thyroid carcinomas **(R)**<br>Pheochromocytoma **(R)**	<u>Pituitary adenomas (prolactinomas, growth hormone, TSH rare, ACTH, nonfunctioning)</u> (25%, unknown the malignant component)<br><br>Adrenocortical Tumors:<br>- Cortical adenomas<br>- Cortical hyperplasia<br>- Multiple adenomas<br>- Nodular hyperplasia<br>- Cysts<br>- Pheochromocytoma **(R)**
Hyperparathyroidsm-jaw tumor syndrome (HPT-JT)	<u>Parathyroid carcinoma</u> (23%)<br>Uterine tumors<br>Wilms tumor **(R)**	<u>Parathyroid adenoma</u><br><u>Jaw tumors: ossifying fibromas of the maxilla and/or mandible (Cementifying fibromas / cemento-ossifying fibromas)</u><br>Kidney cysts, renal hamartoma<br>Benign uterine tumors<br>Mixed epithelial-stromal tumors **(R)**<br>Adult mesoblastic nephroma **(R)**
Parathyroid carcinoma syndrome (PC)	<u>Parathyroid carcinomas (generally secreting)</u> (23%)	None
HOXB13-hereditary cancer syndrome (HOXB13)	<u>Prostate cancer</u> (33%)	None
NBN-hereditary cancer syndrome (NBN)	Breast cancer<br>Prostate cancer<br>Melanoma<br>Medulloblastoma<br>Non-Hodgkin Lymphoma<br>Colorectal cancer	None
Njimegen Breakage Syndrome (NBS)	<u>T cell and B cell lymphomas</u><br>Medulloblastoma<br>Glioma<br>Rhabdomyosarcoma	<u>Disproportionate microcephaly</u><br><u>Craniofacial features ("bird-like face")</u>:<br>- a sloping forehead<br>- upward slanted palpebral fissures<br>- prominent nose<br>- relatively large ears<br>- retrognathia<br><u>Immunodeficiency</u> (agammaglobulinemia) → recurrent infections<br>Autoimmune diseases (e.g., immune thrombocytopenia and hemolytic anemia, childhood sarcoidosis, lymphocytic interstitial pneumonitis)

		Growth retardation and short stature<br>Decline in intellectual ability<br>Hypergonadotropic hypogonadism (in female: primary ovarian insufficiency)<br><br>Skin:<br>Café-au-lait spots<br>Depigmented skin lesions<br>Porokeratosis<br>Cutaneous noncaseating granulomas<br><br>Clinodactyly and syndactyly<br>Choanal atresia<br>Cleft lip and palate<br>Hydronephrosis<br>Gastrointestinal tract stenosis/atresia<br>Hip dysplasia<br><br>Tracheal hypoplasia **(R)**<br>Preaxial or postaxial polydactyly **(R)**<br>Horseshoe kidney **(R)**<br>Hypospadias **(R)**<br>Anomalies of the central nervous system: **(R)**<br>- hydrocephaly<br>- schizencephaly<br>- arachnoid cysts
Von Hippel Lindau Syndrome (VHLS)	Renal cell carcinoma (clear cell subtype) (80%)<br>Pheochromocytoma **(R)** (30%, unknown the malignant percentage)<br>Pancreatic neuroendocrine tumors (12%, unknown the malignant percentage)<br>Endolymphatic sac tumors **(R)** (4%, unknown the malignant percentage)	CNS hemangioblastomas<br>Retinal capillary hemangioblastomas (retinal angiomas)<br>Multiple and bilateral renal cysts<br>Pheochromocytoma<br>Paragangliomas<br>Endolymphatic sac tumors of the middle ear<br>Pancreatic serous cystadenomas<br>Epididymal and broad ligament cystoadenomas<br><br>***VHL type 1****. Retinal angioma, CNS hemangioblastoma, renal cell carcinoma, pancreatic cysts, and neuroendocrine tumors. VHL type 1 is characterized by a low risk for pheochromocytoma.*

		VHL type 2. *Pheochromocytoma, retinal angiomas, and CNS hemangioblastoma. VHL type 2 is characterized by a high risk for pheochromocytoma.*<br>- *Type 2A. Pheochromocytoma, retinal angiomas, and CNS hemangioblastoma; low risk for renal cell carcinoma*<br>- *Type 2B. Pheochromocytoma, retinal angiomas, CNS hemangioblastoma, pancreatic cysts, and neuroendocrine tumors; high risk for renal carcinoma*<br>- *Type 2C. Risk for pheochromocytomas only*
Hereditary papillary renal carcinoma syndrome (HPRC)	Type 1 papillary renal cell cancer (multifocal, bilateral)	
Hereditary leiomyomatosis and renal cancer cell syndrome (HLRCC)	Renal cancer (type 2 papillary renal cell cancer, collecting duct renal cell carcinoma **(R)**, clear cell renal cell carcinoma **(R)**, Wilm's tumor **(R)**) (19%)<br><br>Cutaneous leiomyosarcoma **(R)**<br>Uterine leiomyosarcoma **(R)**<br>Leydig cell tumors **(R)**<br>Gastrointestinal stromal tumors (GIST) **(R)**<br>Breast cancer **(R)**	Cutaneous leiomyomas<br>Uterine leiomyomas<br><br>Ovarian cystadenomas **(R)**<br>Pheochromocytoma **(R)**<br>Paraganglioma **(R)**
Tuberous Sclerosis Complex (TSC)	Brain cancer (malignant transformation of subependymal giant cell tumors) (up to 90%)<br>Renal cell carcinoma, malignant angiomyolipomas (4%)<br>Rhabdomyosarcoma **(R)**	Skin:<br>- Hypomelanotic macules<br>- Confetti skin lesions<br>- Facial angiofibromas<br>- Shagreen patches<br>- Fibrous cephalic plaques<br>- Ungual fibromas<br><br>Central Nervous System:<br>- Cortical glioneuronal hamartomas<br>- Cortical dysplasias<br>- Subependymal nodules (SENs)<br>- Subependymal giant cell astrocytomas (SEGAs)<br><br>Seizures<br>Developmental delay<br>Intellectual disability<br><br>Neuropsychiatric Disorder:

		- Autism spectrum disorder (ASD)<br>- Attention deficit hyperactivity disorder (ADHD)<br>- Learning and cognitive impairment<br>- Disruptive behaviors and emotional problems (anxiety, depression)<br><br>Kidneys:<br>- Benign angiomyolipoma<br>- Lymphangiomas<br>- Renal cysts<br>- Oncocytoma (benign adenomatous hamartoma)<br><br>Cardiac rhabdomyomas<br><br>Lung:<br>- Lymphangioleiomyomatosis (LAM)<br>- Multifocal micronodular pneumonocyte hyperplasia (MMPH)<br><br>Retinal lesions (hamartomas)<br><br>Extrarenal angiomyolipomas (AMLs) (hepatic AMLs)
Birt-Hogg-Dubé syndrome (BHD)	Kidney cancer (19%)<br>- Hybrid oncocytic/chromophobe tumor<br>- Chromophobe tumor<br>- Oncocytoma<br>- Clear cell carcinoma<br>- Papillary carcinoma **(R)**<br>- Mixed-type carcinoma **(R)**<br><br>Colon cancer **(R)**<br>Cutaneous melanoma (including multiple desmoplastic melanomas) and choroidal melanoma **(R)**<br>Parotid **(R)**<br>Thyroid cancer **(R)**<br>Parathyroid cancer **(R)**<br><br>Basal cell carcinoma **(R)**	Cutaneous manifestations:<br>- Fibrofolliculomas<br>- Skin tags (acrochordons)<br>- Angiofibromas<br>- Oral papules<br>- Cutaneous collagenomas<br>- Multiple epidermal cysts<br>- Koenen's tumor **(R)**<br>- Trichoblastoma<br><br>Soft tissues:<br>- Angiolipoma **(R)**<br>- Leiomyoma **(R)**<br>- Lipoma **(R)**<br>- Fibroadenomatosis **(R)**

	Squamous cell carcinoma **(R)**<br>Dermatofibrosarcoma protuberans **(R)**<br>Sarcomas **(R)**<br>Gastric cancer **(R)**<br>Peritoneal mesothelioma **(R)**<br>Head & neck cancer **(R)**<br>Oncocytic adrenal tumor **(R)**<br>Lymphoma & leukemia **(R)**<br>Lung cancer **(R)**<br>Neuroendrocine tumors **(R)**<br>Prostate cancer **(R)**<br>Breast cancer **(R)**<br>Uterine cancer **(R)**	Muscoskeletal:<br>- Cardiac rhabdomyoma **(R)**<br>- Osteoma **(R)**<br>- Rhabdomyoma **(R)**<br><br>Lungs:<br>- <u>Lung cysts</u><br>- Spontaneous pneumothorax<br>- Clear cell sugar tumor **(R)**<br><br>Multifocal renal oncocytosis<br>Renal angiomyolipoma **(R)**<br><br>Colon polyps **(R)**<br><br>Warthin parotid tumor<br>Thyroid nodules/cysts<br>Parathyroid adenoma **(R)**<br>Adrenal adenoma **(R)**<br>Pheochromocytoma **(R)**<br>Hepatic cysts or angioma **(R)**<br><br>Nervous system:<br>- Astrocytoma **(R)**<br>- Cerebral hemangioma **(R)**<br>- Meningioma **(R)**<br>- Neurothekeoma **(R)**<br>- Oncocytic pituitary adenoma **(R)**<br>- Schwannoma **(R)**
Li-Fraumeni syndrome (LF)	<u>Adrenocortical carcinomas</u> (ACC) (.7%)<br><u>Breast cancer</u> (ductal, ER+, PR+, HER2+, malignant phyllodes tumors of the breast) (85% in female)<br><u>Nervous system cancer</u> (glioblastoma, astrocytoma, ependymoma, medulloblastoma, supratentorial primitive neuroectodermal tumors, choroid plexus carcinomas) (30%))<br><u>Sarcomas</u> (osteosarcoma, soft-tissue sarcoma (e.g., rhabdomyosarcoma)) (60%)	Colorectal polyps

	Hematologic cancer (leukemia (ALL, AML), myelodysplastic syndrome (MDS), Hodgkin and non-Hodgkin lymphomas)<br>Gastrointestinal cancer (colorectal cancer up to 24%, gastric cancer up to 16%)<br>Cancers of the head and neck<br>Kidney cancers<br>Lung cancers<br>Skin cancers<br>Ovary cancers<br>Pancreas cancers<br>Testis cancers<br>Thyroid cancers<br>Gestational choriocarcinoma (The pregnant mother of a fetus heterozygous for a paternally inherited TP53 pathogenic variant is at risk for choriocarcinoma or another gestational trophoblastic disease)	
Bloom syndrome (BSyn)	Leukemia (AML, ALL)<br>Non-Hodgkin lymphoma<br>Myelodysplasia<br><br>Oropharyngeal tumors<br>Upper GI tumors<br><br>Colorectal carcinoma<br><br>Genitourinary tumors<br>Skin carcinomas<br>Breast carcinoma<br><br>Wilms tumor<br>Lung tumors	Habitus:<br>- Proportionate small stature<br>- Mild microcephaly<br>- Keel-shaped face<br>- Malar hypoplasia<br>- Nasal prominence<br>- Small mandible<br>- Protuberant ears<br>- Absence of upper lateral incisors<br><br>Skin:<br>- Sparse, subcutaneous adipose tissue<br>- Erythematous rash on the nose and cheeks (sun-sensitive facial erythema)<br>- Café-au-lait spots<br>- Hypopigmented skin lesions<br>- Cheilitis<br>- Blistering and fissuring of the lips<br>- Eyebrow and eyelash hair loss<br>- Alopecia areata<br>- Vesicular and bullous lesions<br><br>Infertility in men (hypogonadism)<br>Premature menopause in women (premature menopause)

		Immunodeficiency<br>Variable intellectual impairment<br>Diabetes mellitus<br>Gastroesophageal reflux<br>Chronic obstructive pulmonary disease
Familial GIST (fGIST)	GISTs	Hyperpigmentation (KIT)<br>Urticaria pigmentosa (KIT)<br>Dysphagia (KIT)<br>Gastrointestinal autonomic nerve tumors (paragangliomas) (KIT)<br>Intestinal fibromatosis (PDGFRA)<br>Inflammatory fibroid polyps (PDGFRA)<br>Large hands (PDGFRA)
BRCA1- and BRCA2-associated hereditary cancer syndrome (BRCA1/2)	Breast cancer (in females: 72% BRCA1, 69% BRCA2, in males: 1.2% BRCA1, 7% BRCA2)<br>Ovarian cancer (44% BRCA1, 17% BRCA2)<br>Fallopian tube and primary peritoneal cancers **(R)** (2% BRCA1, 1% BRCA2)<br>Prostate cancer (29% BRCA1, 60% BRCA2)<br>Pancreatic cancer (16% BRCA1, 13.7% BRCA2)<br>Gastric cancer (21.3% BRCA1, 19.3% BRCA2)<br>Biliary tract (11.2% BRCA1)<br>Esophageal (5.2% BRCA2) **(R)**<br>Cutaneous and ocular melanoma (BRCA2) **(R)**<br>Colon cancer **(R)**<br>Lung cancer **(R)**	Ovarian cysts
CHEK2-associated hereditary cancer syndrome (CHEK2)	Breast cancer (30%)<br>Colon cancer **(R)**<br>Prostate cancer **(R)**<br>Gastric cancer **(R)**<br>Renal cancer **(R)**<br>Thyroid cancer **(R)**<br>Sarcoma **(R)**<br>Leukemia and plasma cell neoplasm **(R)**	
PALB2-associated hereditary cancer syndrome (PALB2)	Breast cancer (53%)<br>Pancreatic cancer (3%)<br>Ovarian cancer **(R)** (5%)	
RAD51c-associated hereditary cancer syndrome (RAD51c)	Ovarian cancer (11%)<br>Breast cancer (21%)	

RAD51d-associated hereditary cancer syndrome RAD51d)	Ovarian cancer (13%)<br>Breast cancer (20%)	
ATM-associated hereditary cancer syndrome (ATM)<br>Ataxia telangiectasia (AT)	**Heterozygosis**<br>Pancreatic cancer (9.5%)<br>Prostate cancer<br>Gastric cancer<br>Breast cancer (30%, c.7271T>G variant 52%)<br>Male breast cancer<br>Ovarian cancer<br>Melanoma<br>Lymphoma/leukemia<br><br>**Homozygosis – classical Ataxia-teleangectasia**<br>Lymphoma/leukemia (32%)<br>Ovarian cancer **(R)**<br>Breast cancer **(R)**<br>Liver cancer **(R)**<br>Gastric cancer **(R)**<br>Esophageal cancer **(R)**<br>Sarcomas **(R)**	**Heterozygosis**<br>Coronary heart disease<br><br>**Homozygosis – classical Ataxia-teleangectasia**<br>• Serum and cellular immunodeficiency with frequent infections (recurrent sinopulmonary infections)<br><br>Neurological deficits:<br>• Progressive cerebellar ataxia with onset between ages one and four years, oculomotor apraxia, choreoathetosis<br>• Progressively slurred speech<br>• Extrapyramidal movement disorders<br>• Peripheral neuropathy<br><br>Telangiectasias and skin abnormalities<br>• Oculocutaneous telangiectasias<br>• Skin telangiectasias (pinnae, nose, face, neck)<br>• Other (cafè-au-lait macules, hypopigmented macules, melanocytic nevi, facial papulosquamous rash)<br>• Premature aging with strands of gray hair<br><br>• Pulmonary disease (bronchiectasis, interstitial lung disease/pulmonary fibrosis)<br><br>• Endocrine alterations (gonadal dysgenesis, type 2 diabetes mellitus)<br>• Laboratory abnormalities (increase AFP, T cell lymphopenia, immunoglobulin deficiency<br><br>**Non classic form of AT**<br>Attenuated phenotype
STK11-associated hereditary cancer syndrome (STK11)	Colorectal cancer (39%)<br>Gastric cancer (29%)<br>Small bowel cancer (13%)<br>Pancreatic cancer (26%)<br>Breast cancer (32-54%)<br>Ovarian cancer (mostly sex cord tumor with annular tubules) (21%)	Multiple gastrointestinal hamartomatous polyps and in extraintestinal sites (bronchus, nasal passages, gall bladder, renal pelvis, urinary bladder, and ureters).<br><br>Multiple melanocytic macules in lips, buccal mucosa, and digits.

	Adenoma malignum of the cervix (10%)<br>Uterus cancer (9%)<br>Sertoli cell tumors (9%)<br>Lung (7-17%)	Men: occasionally large calcifying Sertoli cell tumors (LCST) of the testes, with consequent gynecomastia, advanced skeletal age, and ultimately short stature, due estrogen secretion
BARD1-associated hereditary cancer syndrome (BARD1)	<u>Breast cancer (15-40%)</u><br>Colon cancer **(R)**<br>Ovarian cancer **(R)**	
BRIP1- associated hereditary cancer syndrome (BRIP1)	Ovarian cancer (4-13%)<br>Breast cancer **(R)**	
Fanconi Anemia	<u>Myelodysplastic syndrome/leukemia (80%)</u><br>Lymphoid malignancies (acute lymphoid leukemia, Burkitt lymphoma) **(R)**<br><br>Skin cancer (squamous cell carcinoma, basal cell carcinoma)<br>Head & neck cancer (squamous cell carcinoma)<br>Esophageal cancer<br>Vulvar cancer<br>Anal cancer (squamous cell carcinoma)<br>Liver cancer<br>Kidney cancer<br>Brain cancer<br>Breast cancer<br>Testis/ovarian cancer (germ cell tumors) **(R)**<br>Sarcomas **(R)**<br><br>**PALB2/BRCA2**<br>Medulloblastoma<br>Wilms tumor	<u>Classical congenital abnormalities the VACTERL-H</u> (Vertebral, Anal, Cardiac, Tracheo-esophageal fistula, Esophageal atresia, Renal, upper Limb and Hydrocephalus) association.<br><br><u>Physical Findings:</u><br>• Endocrine disorders: hypothyroidism, hyperglycemia/ impaired glucose tolerance, diabetes, and insulin resistance<br>• Skin pigmentation alterations: café au lait macules, generalized hyperpigmentation, hypopigmentation<br>• Growth deficiency: prenatal and/or postnatal short stature, low birth weight<br>• Skeletal malformations of upper limbs, unilateral or bilateral:<br>o Thumbs: absent, triphalangeal, proximally placed, bifid, duplicated, long, hypoplastic<br>o Radii: absent or weak pulse, absent or hypoplastic (only with abnormal thumbs)<br>o Hands: absent first metacarpal, flat thenar eminence, polydactyly, clinodactyly<br>o Ulnae: short, dysplastic<br>o Lower limbs: congenital hip dislocation, syndactyly, abnormal toes, club feet<br>o Facial features: mid-face hypoplasia, triangular face shape, micrognathia<br>o Spine anomalies: spina bifida, scoliosis, hemivertebrae, rib anomalies, coccygeal aplasia<br>o Neck anomalies: sprengel deformity, Klippel-Feil anomaly, short or webbed neck, low hairline<br>• Microcephaly

		• Ophthalmic alterations: strabismus, microphthalmia, hypertelorism, ptosis, cataracts, astigmatism, epicanthal folds, hypotelorism<br>• Developmental Delay / Intellectual Disability<br>• Genitourinary tract anomalies:<br>o Rena: hypoplastic, dysplastic, horseshoe, ectopic, pelvic, or absent kidney; hydronephrosis or hydroureter<br>o Males: hypospadias, cryptorchidism, anorchia, micropenis, hypo- or azoospermia, reduced fertility<br>o Females: small ovaries, bicornuate or uterus malposition<br>• Hearing loss. Usually conductive secondary to middle-ear bony anomalies with or without additional ear anomalies (e.g., dysplastic auricle, narrow ear canal, abnormal pinna)<br>• Congenital heart defect: atrial septal defect, patent ductus arteriosus, coarctation of the aorta, ventricular septal defect, truncus arteriosus, situs inversus<br>• Gastrointestinal: imperforate anus, tracheoesophageal fistula, esophageal, duodenal, or jejunal atresia, annular pancreas, malrotation<br>• Central nervous system: cerebellar hypoplasia, hydrocephalus, dilated ventricles, small pituitary, pituitary stalk interruption syndrome, absent corpus callosum<br><br><u>Bone Marrow Failure</u>:<br>• Thrombocytopenia or leukopenia usually precede anemia, commonly associated with macrocytosis and elevated fetal hemoglobin<br>• Pancytopenia<br>• Sweet syndrome (neutrophilic skin infiltration)<br>• Bone marrow failure
Familial atypical multiple mole melanoma (FAMMM)	<u>Melanoma (28-67%)</u><br>Pancreatic cancer (adenocarcinoma) (17%)<br>Astrocytoma (loss of both p14 and p16)<br>Breast cancer **(R)**<br>Lung, head & neck, gastroesophageal cancer (in smokers) **(R)**	Total body nevi count > 50<br>Multiple atypical nevi
Nevoid basal cell carcinoma (NBCC)	<u>Basal cell carcinomas (90%)</u><br>Medulloblastoma (SUFU 33% - PTCH1 <2%)<br>Rhabdomyosarcoma **(R)**	Jaw odontogenic keratocyst<br>Palmar and plantar pits<br>Intracranial ectopic calcification<br>Meningiomas

		Craniofacial anomalies (macrocephaly, frontal bossing, hypertelorism)<br>Vertebral/rib anomalies<br>Cleft lip/palate<br>Polydactyly<br>Cardiac or ovarian fibromas<br>Lymphomesenteric or pleural cysts<br>Ocular anomalies (congenital cataracts, coloboma, strabismus, hyperthelorim, telecanthus)<br>Fetal rhabdomyoma
Xenoderma pigmentosus (XP)	<u>Skin cancer (basal cell carcinoma, squamous cell carcinoma, melanoma) (65%)</u><br>Oral cancer<br>Sarcoma (angiosarcoma, fibrosarcoma, and pyogenic granuloma of oral cavity)<br>Myelodysplastic syndrome, acute myeloid leukemia, and acute lymphoblastic leukemia **(R)**<br>Colorectal cancer **(R)**<br>Non-small cell lung cancer **(R)**<br>Glioma **(R)**<br>Uterine cancer **(R)**<br>Breast cancer **(R)**<br>Pancreatic cancer **(R)**<br>Stomach cancer **(R)**<br>Kidney cancer **(R)**<br>Testicle cancer **(R)**<br>Thyroid cancer **(R)**	<u>Sun sensitivity (severe sunburn with blistering, persistent erythema, xerosis, and poikiloderma on minimal sun exposure, Sunlight-induced ocular involvement such as photophobia, keratitis, atrophy of the skin of the lids)</u><br><br>Eye anomalies (ectropion, lagophtalmos, conjunctival melanosis, corneal neovascularization, corneal scarring, prerygium)<br><br>Marked freckle-like pigmentation of the face<br><br>Neurologic manifestations (progressive sensorineural hearing loss, acquired microcephaly, progressive cognitive impairment, and diminished or absent deep tendon stretch reflexes)<br><br>Premature menopause
BAP1 tumor predisposition syndrome (BAP1)	<u>Uveal melanoma (36%)</u><br><u>Cutaneous melanoma (12-45%)</u><br>Malignant mesothelioma (25%)<br>Renal cell carcinoma (10%)<br>Basal cell carcinoma<br>Cholangiocarcinoma **(R)** (1.4%)<br>Hepatocellular carcinoma **(R)** (0.7%)<br>Breast cancer **(R)**<br>Lung cancer **(R)**<br>Sarcoma **(R)**<br>Neuroendocrine tumors **(R)**	Multiple cutaneous melanocytic neoplasms called "BAP1-inactivated melanocytic tumors" (BIMTs; melanocytomas)<br><br>Meningioma

	Thyroid cancer **(R)**	
Shelterin complex genes hereditary cancer syndrome (Shelterin)	Chronic lymphocytic leukemia<br>Glioma<br><u>Melanoma</u><br>Angiosarcoma (cardiac angiosarcoma)<br><br>Colorectal cancer **(R)**<br>Thyroid cancer **(R)**	None
TERT hereditary cancer syndrome (TERT)	<u>Acute myeloid leukemia/chronic lymphocytic leukemia</u><br>Cutaneous melanoma<br>Pancreatic cancer<br>Lung cancer<br>Basal cell carcinoma<br>Head & neck cancer<br>Anogenital cancer<br>Bladder cancer **(R)**<br>Prostate cancer **(R)**<br>Cervical cancer **(R)**<br>Glioma **(R)**	Dyskeratosis congenita:<br>Developmental delay, short stature, microcephaly, osteoporosis, avascular necrosis of femur/humerus<br>Blepharitis, epiphora<br>Periodontal disease, taurodontism, decreased teeth/root ratio<br>Esophageal stenosis, urethral stenosis<br>Premature hair greying/alopecia, abnormal eyelashes<br>Pulmonary fibrosis, pulmonary arteriovenous malformations<br>Gastrointestinal teleangiectasias<br>Liver disease
MC1R polymorphism (MC1R)	<u>Cutaneous melanoma</u><br>Squamous cell carcinoma **(R)**<br>Basal cell carcinoma **(R)**	Red hair/fair skin
MITF (E318K) polymorphism	<u>Cutaneous melanoma</u><br>Uterine carcinosarcoma **(R)**<br>Renal cancer **(R)**<br>Pheochromocytoma/paraganglioma **(R)**<br>Pancreatic cancer **(R)**	None
DICER1	<u>Pleuropulmonary blastoma</u> (up to 50%)<br>Ovarian sex cord-stromal tumor (10%)<br>Medulloepithelioma (3%)<br>Differentiated thyroid carcinoma **(R)**<br>Embryonal rhabdomyosarcoma of the cervix **(R)**<br>Centran nervous system cancers (pituitary blastoma, pineoblastoma, sarcomas, presacral malignant teratoid tumor, other embryonal tumors) **(R)**	Macrocephaly<br>Retinal abnormalities<br>Multinodular goiter<br>Cystic nephroma<br>Abnormal of the collecting system of the kidney<br>Nasal chondromesenchymal hamartoma<br>Dental anomalies
Lynch syndrome (LS),	<u>Colorectal cancer (CRC)</u> (MLH1 44-53%, MSH2 42-46%, MSH6 12-20%, PMS2 3%, EPCAM 75%)	Skin tumors (sebaceous neoplasms, keratoacanthomas)

Hereditary nonpolyposis colorectal cancer (HNPCC)	<u>Endometrial cancer (EC)</u> (MLH1 35%, MSH2 46%, MSH6 41%, PMS2 13%, EPCAM 12%)<br>Ovarian cancer (endometrioid) (MLH1 11%, MSH2 17%, MSH6 11%, PMS2 3%)<br>Gastric cancer (GC) (mostly intestinal-type adenocarcinoma, rarely diffuse type) and small bowel cancer (duodenum and jejunum adenocarcinomas) (MLH1 8-16%, MSH2 10-16%, MSH6 2-4%, PMS2 4%)<br>Urinary tract cancer (transitional carcinomas of the ureter, renal pelvis, kidney, and bladder cancer) (MLH1 5%, MSH2 16%, MSH6 6%)<br>Skin cancers (sebaceous carcinomas)(<9%)<br>Pancreatic cancer<br>Prostate cancer (MLH1 7%, MSH2 16%, MSH6 5%, PMS2 5%)<br>Brain (usually glioblastoma; astrocytoma, oligodendroglioma)(1-4%)<br>Breast cancer **(R)**<br>Adrenocortical carcinomas **(R)**<br>Laryngeal cancer **(R)**<br>Hematologic malignancies **(R)**<br>Sarcomas **(R)**	Adenomas of the colon (mostly with high grade dysplasia and/or villous histology)
RPS20-associated hereditary nonpolyposis colorectal cancer syndrome (RPS20)	<u>Colorectal cancer</u>	
Familial adenomatous polyposis (FAP)	<u>Colorectal cancer (often <40)</u> (>95%)<br>Duodenal and periampullary cancer (<12%)<br>Brain tumors (Turcot syndrome): medulloblastoma, ependymomas and high-grade astrocytoma (1%)<br>Thyroid papillary carcinoma (<12%)<br>Hepatoblastoma (<1%)<br>Pancreatic cancer (1%)<br>Gastric cancer (1%)<br>Desmoid tumors (30%)	<u>Adenomatous polyposis (>100 colon and rectum polyps)</u><br><br>Extracolonic manifestations (Gardner syndrome):<br>- Gastric fundic gland polyps<br>- Duodenal adenomas<br>- <u>Congenital hypertrophy of the retinal pigment epithelium (CHRPE)</u><br>- Juvenile nasopharyngeal angiofibromas<br>- Osteomas, fibromas<br>- Dental abnormalities: supernumerary teeth, odontomas<br>- Epidermoid or sebaceous cysts<br>- Benign thyroid nodules
Attenuated familial adenomatous polyposis (AFAP)	<u>Colorectal cancer (> 40 years)</u><br>Thyroid cancer<br>Duodenal cancer	<u>Adenomatous polyposis (10-100 polyps, frequently right sided)</u><br>Upper GI findings similar to FAP<br><br>Other extraintestinal manifestations are unusual:

		- <u>Congenital hypertrophy of the retinal pigment epithelium (CHRPE)</u> **(R)**<br>- Juvenile nasopharyngeal angiofibromas **(R)**<br>- Osteomas, fibromas **(R)**<br>- Dental abnormalities: supernumerary teeth, odontomas **(R)**<br>- Desmoid tumors **(R)**<br>- Epidermoid or sebaceous cysts **(R)**<br>- Benign thyroid nodules **(R)**
Gastric adenocarcinoma and proximal polyposis of the stomach (GAPPS)	<u>Gastric cancer (intestinal gastric type adenocarcinoma)</u>	<u>Gastric fundic gland polyps (restricted to body and fundus of the stomach)</u>
Polymerase proofreading-associated polyposis (PPAP)	<u>Colorectal cancer (mostly MSS)</u> (30-60%)<br>Duodenal cancer<br>Endometrial cancer<br>Ovarian cancer<br>Brain cancer<br>Breast cancer	<u>Colonic adenomas (0-100 cumulative polyps)</u><br>Duodenal adenomas<br>MDPL syndrome (mandibular hypoplasia, deafness, progeroid features & lipodystrophy) (POLD1)
MUTYH associated polyposis (MAP)	<u>Colorectal cancer (mostly MSS)</u> (86%)<br>Duodenal cancer (4%)<br>Ovarian cancer (14%)<br>Bladder cancer (6-25%)<br>Endometrial cancer (3%)<br>Gastric cancer (R) (1%)<br>Kyndey cancer **(R)**<br>Breast cancer **(R)** (<25%)<br>Skin cancer **(R)**<br>Thyroid cancer **(R)**<br>Pancreatic cancer **(R)**	<u>Colonic adenomas (10->100 cumulative polyps)</u><br>Colonic hyperplastic and/or serrated polyps<br>Gastro-duodenal adenomas<br>Benign adrenal lesions<br>Thyroid nodules<br>Jawbone cysts<br>Osteomas<br>Congenital hypertrophy of the retinal pigment epithelium (CHRPE)<br>Desmoid tumors<br>Epidermoid or sebaceous cysts
NTHL1 tumor syndrome (NTHL1)	<u>Colorectal cancer</u><br>Breast cancer<br>Duodenal cancer<br>Endometrial cancer<br>Cervical cancer<br>Urothelial carcinoma of the bladder<br>Brain cancer<br>Basal cell carcinoma<br>Head and neck squamous cell carcinomas<br>Hematologic malignancies	<u>Colonic adenomas (1-100 cumulative polyps)</u><br>Hyperplastic and/or serrated polyps<br>Duodenal adenomas<br>Meningiomas<br>Skin hemangiomas **(R)**<br>Seborrheic keratosis **(R)**, intradermal nevi **(R)**<br>Ovarian and hepatic cysts **(R)**<br>Breast papillomas **(R)**

MSH3-associated polyposis (MSH3)	Colorectal cancer<br>Gastric cancer<br>Astrocytoma	Colonic adenomas (10->100 cumulative polyps)<br>Duodenal adenomas<br>Thyroid adenomas<br>Mammary intraductal papillomas and cysts
MLH3-associated polyposis (MLH3)	Colorectal cancer<br>Breast cancer	Colonic adenomas (10->100 cumulative polyps)
Juvenile polyposis syndrome (JPS)	Colorectal cancer (40%)<br>Gastric cancer (21%)<br>Small bowel cancer **(R)**<br>Pancreatic cancer **(R)**	Hamartomatous gastrointestinal polyps<br>Hereditary hemorrhagic telangiectasia or vascular abnormalities<br>Cardiac abnormalities<br>Skeletal and cranial abnormalities
Hereditary mixed polyposis syndrome (HMPS)	Colorectal cancer<br>Duodenal adenocarcinoma **(R)**<br>Prostate cancer **(R)**	Adenomatous polyps, juvenile polyps, hyperplastic polyps, and polyps containing mixed histology<br>Desmoid tumor **(R)**
RNF43-associated serrated polyposis (RNF43), Sessile serrated polyposis cancer syndrome (SSPCS)	Colorectal cancer	Colonic serrated polyposis (0->100 cumulative polyps)
Cowden Syndrome (CS)	Breast cancer (30%)<br>Epithelial thyroid cancer (usually follicular, rarely papillary, never medullary) (>10%)<br>Genitourinary cancer (especially renal cell carcinoma) (10%)<br>Endometrial cancer (10%)<br>Colorectal cancer **(R)** (9%)<br>Melanoma **(R)** (6%)	Cerebellar dysplastic gangliocytoma (Adult Lhermitte-Duclos disease)<br>Intracranial vascular malformations<br>Macrocephaly, intellectual disabilities, autism<br><br>Mucocutaneous lesions: trichilemmomas (facial), acral keratoses, papillomatous lesions, mucocutaneous neuromas<br>Benign lesions of thyroid (adenoma, multinodular goiter)<br><br>Colorectal and gastrointestinal polyps (often hamartomas)<br>Esophageal glycogen acanthosis<br><br>Fibrocystic disease of the breast<br>Lipomas, fibromas, macular pigmentation of the glans penis<br>Genitourinary malformation<br>Ttesticular lipomatosis<br>Uterine fibroids
Hereditary diffuse gastric cancer syndrome (HDGC)	Gastric cancer (signet ring cancer/diffuse gastric cancer) (33-70%)<br>Breast cancer (lobular histology) (42%)<br>Colorectal cancer **(R)**	Cleft lip/palate **(R)**

Hereditary pancreatitis (HP)	Pancreatic cancer	Acute and chronic pancreatitis<br><br>Sinusitis, respiratory symptoms, male infertility, constipation (CFTR)
Howel-Evans syndrome (HE)	Esophageal cancer (squamous cell carcinoma)	Palmoplantar keratoderma (tylosis)<br>Leukoplakia
EGFR-associated genetic susceptibility	Lung cancer	

Supplementary Table S1. Detailed list of features associated with hereditary cancer syndromes. Underlined = a typical feature; (R) = rarely associated or weakly supported by scientific literature; (xxx%) = lifetime risk. Sources: Pubmed, Web of Science, GeneReview, UptoDate, Omim, EviQ Genetics.
